# Multiorgan Molecular Landscape of Severe COVID‐19 Revealed by Consensus Gene Signatures and RAB8B Targeting

**DOI:** 10.1002/jmv.70932

**Published:** 2026-04-21

**Authors:** Jonathan Peña Avila, Peter Park, Youvika Singh, Paulo P. Amaral, Ícaro Castro, Felipe Ten‐Caten, Viviane Schuch, André N. A. Gonçalves, Jeevan Giddaluru, Mauro César Cafundó Morais, Rodrigo L. T. Ogava, Thiago Lubiana, Gabriel Amoroso de Castro, Rodrigo Aquino, Luiz Durão, Júlia Raspante Martins, Leandro Jimenez, André G. Costa‐Martins, Patrícia Gonzalez‐Dias, Thiago Dominguez Crespo Hirata, Thomaz Lüscher Dias, Débora Guerra Peixe, Adriana Simizo, Juan Carlo Santos e Silva, Amanda Pereira Vasconcelos, Marcelo Berçot Rodrigues, Bianca G. Castelucci, João Victor Virgillio‐da‐Silva, Larissa Menezes, Pedro M. Moraes‐Vieira, Otavio Cabral‐Marques, Helder I. Nakaya

**Affiliations:** ^1^ Department of Clinical and Toxicological Analyses University of São Paulo São Paulo São Paulo Brazil; ^2^ Institut Pasteur de São Paulo University of São Paulo São Paulo São Paulo Brazil; ^3^ Department of Parasitology Leiden University Center for Infectious Diseases (LU‐CID), Leiden University Medical Center Leiden The Netherlands; ^4^ Instituto de Ensino e Pesquisa, Insper São Paulo Brazil; ^5^ Interunit Postgraduate Program on Bioinformatics, Institute of Mathematics and Statistics University of São Paulo São Paulo São Paulo Brazil; ^6^ Department of Infectious and Parasitic Diseases University of São Paulo Medical School, University of São Paulo São Paulo Brazil; ^7^ Department of Pharmaceutical Sciences State University of Campinas Campinas Brazil; ^8^ Department of Pharmaceutical Sciences University of São Paulo Ribeirão Preto São Paulo Brazil; ^9^ Micro Manufacturing Laboratory, Institute for Technological Research – IPT São Paulo São Paulo Brazil; ^10^ Hospital Israelita Albert Einstein São Paulo São Paulo Brazil; ^11^ Department of Genetics, Microbiology and Immunology, Institute of Biology State University of Campinas Campinas Brazil; ^12^ Department of Immunology, Institute of Biomedical Sciences University of São Paulo São Paulo São Paulo Brazil; ^13^ Laboratory of Medical Investigation, Department of Medicine University of São Paulo School of Medicine, Division of Molecular Medicine São Paulo São Paulo Brazil; ^14^ Instituto D'Or de Ensino e Pesquisa São Paulo Brazil; ^15^ Network of Immunity in Infection, Malignancy, Autoimmunity (NIIMA), Universal Scientific Education and Research Network (USERN) São Paulo São Paulo Brazil

**Keywords:** bulk RNA‐seq, gene expression, RAB8B, SARS‐CoV‐2, scRNA‐seq, severe COVID‐19

## Abstract

Severe COVID‐19 involves hyperinflammation and multiorgan pathology, but consistent gene signatures remain elusive. We aimed to identify consensus transcriptomic signatures and molecular mechanisms in severe COVID‐19. We performed an integrative analysis of 39 studies spanning 11 tissue types, 1551 bulk RNA‐seq samples, and over 2 million single cells. A vote‐counting strategy combined with a systems‐biology approach was applied to detect consensus differentially expressed genes (DEGs). Pathways related to interferon/TNF‐α signaling, hypoxia response, and platelet activation were consistently enriched across data sets. Among consensus DEGs—such as IFITM3, BCL2A1, CAMK2D, and CCR1—RAB8B was prioritized for functional validation based on its recurrence in ~45% of tissues and its known role in vesicle trafficking, a process intimately linked to viral life cycles. Molecular dynamics simulations and in vitro assays in SARS‐CoV‐2‐infected CaCo‐2 cells demonstrated that RAB8B modulates VAMP‐3 clustering and intracellular trafficking. Silencing of Rab8b‐1 and Rab8b‐2 reduced viral infection by 30% (*p* = 0.0302) and 76% (*p* < 0.001), respectively. This study defines robust consensus signatures and positions RAB8B as a critical host factor and potential therapeutic target in severe COVID‐19. Further exploration of RAB8B inhibitors is warranted to explore therapeutic utility. An interactive database at https://covidatlas.sysbio.tools/.

## 1 Introduction

The COVID‐19 pandemic, caused by the SARS‐CoV‐2 virus, has emerged as an unparalleled global health crisis, affecting millions with a broad spectrum of clinical outcomes. This spectrum ranges from mild symptoms like coughing to severe conditions, including multiorgan failure, underlining the profound and diverse impact of the virus [1]. Predominantly, Acute Respiratory Distress Syndrome is a critical factor in mortality among severe cases [2]. Nevertheless, a broader systemic inflammatory response affecting multiple organs is increasingly evident [3]. However, the underlying factors driving this severe disease progression remain complex and uncertain.

Severe COVID‐19 is characterized by a pathogenesis marked by a hyperinflammatory state, lymphopenia, neutrophilia, and elevated levels of acute phase reactants [4], T cell exhaustion [5], and B cell dysregulation [6]. These factors collectively contribute to the development of multiorgan failure and an increased risk of mortality in affected individuals [7, 8]. This state is further associated with a lowered production of interferon (IFN) response against SARS‐CoV‐2 and increased proinflammatory responses involving cytokines such as IL‐6 and TNF‐α [9, 10, 11]. Furthermore, the role of cytokines including TGF‐β, IL‐10, and IL‐8 in severe COVID‐19 cases is increasingly recognized [12, 13].

Advancements in bulk RNA sequencing (bulk RNA‐seq) and single‐cell RNA sequencing (scRNA‐seq) have been instrumental in dissecting COVID‐19's transcriptomic landscape, revealing both shared and distinct molecular disruptions across tissues [14, 15, 16]. For instance, tissue‐specific alterations include solute carrier family proteins in the liver and kidney, as well as troponin‐related genes in the heart [14]. Despite these advances, a consistent picture is yet to emerge, as exemplified by conflicting reports on the regulation of type I interferon (IFN‐I) genes in lung samples from COVID‐19 patients. While one study reports upregulation of IFN‐I genes [14, 17] another suggests a low IFN‐I response induced by SARS‐CoV‐2 infection [18]. While several integrative studies have attempted to address these discrepancies, there remains a gap in integrating transcriptomic data from different tissues and cell types [19, 20, 21]. To overcome these limitations and build a comprehensive understanding, our study employs a robust integrative approach.

Among these complexities, our study aims to identify consensus gene signatures and conserved molecular pathways across multiple organs in severe COVID‐19 using integrated bulk and single‐cell RNA‐seq analysis. By performing a comprehensive analysis of 1551 bulk RNA‐seq samples and over 2 million cells from scRNA‐seq from 14 organs of severe COVID‐19 patients, we not only validate known pathways, such as neutrophil and platelet degranulation but also unveil novel insights in the disease's pathology. Our findings offer a panoramic view of the molecular landscape of severe COVID‐19, providing invaluable insights for researchers and clinicians in navigating the challenges of this health crisis. This sets the stage for the development of targeted therapeutic strategies.

## 2 Results

### 2.1 Data Collection and Consensus Gene Signatures Identification

**Figure 1:**
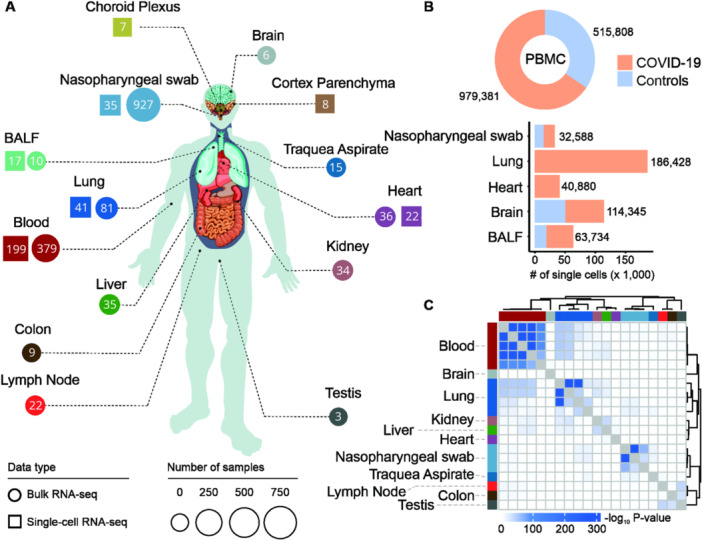
Transcriptome data and consensus gene signatures. (A) Schematic representation of the human body illustrates the distribution of samples across diverse tissues and data types. Each tissue is annotated with its corresponding sample count. We selected 39 studies (14 scRNA‐seq, 24 bulk RNA‐seq, and 1 combining both techniques; Supporting Information S2: Table 1) based on strict inclusion and exclusion criteria. This work incorporated data from 3026 patients: 2493 from bulk RNA‐seq studies across 11 tissues and 533 from scRNA‐seq studies covering 6 tissues. (B) Donut plot and bar chart display the cell count for scRNA‐seq data from peripheral blood mononuclear cells (PBMC) and other tissues, categorized by control and COVID‐19 status, respectively. The scRNA‐seq studies involved the analysis of ~2 042 512 cells. (C) Heatmap represents the Fisher test enrichment scores (−log10padj). Rows and columns represent lists of consensuses differentially expressed genes in bulk RNA‐seq, demonstrating tissue‐specific clustering. The analysis elucidates the robustness of the consensus gene signatures.

We initiated our study with a comprehensive literature review, identifying 112 transcriptome studies. After applying stringent inclusion and exclusion criteria, we selected 39 studies for in‐depth analysis, incorporating both bulk and single‐cell RNA‐seq data sets (Supporting Information S2: Table 1). The integrated data set included samples from over 3000 patients, spanning 11 tissues for bulk RNA‐seq and 6 tissues for scRNA‐seq (Figure 1A). Notably, the scRNA‐seq data alone comprised more than 2 million cells (Figure 1B).

Our next objective was to identify consensus gene signatures and evaluate their consistency. Consensus differentially expressed genes (DEGs) from bulk RNA‐seq were identified using a vote‐counting strategy combined with a systematic iterative pathway enrichment analysis. We calculated the median log2FC and combined *p* values using Fisher's method (Section 5.3).

By comparing consensus DEGs with the original DEGs from each tissue, we observed distinct clustering patterns, indicating a high degree of gene overlap across tissues. This overlap reflects shared biological signals rather than random variation, demonstrating the reliability and consistency of the consensus DEGs (Figure 1C). In contrast, when all DEGs from the original studies were used, this clustering was absent (Supporting Information S1: Figure 3A), further supporting the robustness of our consensus‐based approach for identifying gene signatures specific to severe COVID‐19.

### 2.2 Identification of Essential Genes in Severe COVID‐19 Across Different Tissues

Our bulk RNA‐seq analysis unveiled 9449 consensus DEGs across 11 tissues (Supporting Information S3: Table 2). Notably, peripheral blood emerged as a critical site of systemic involvement in severe COVID‐19, with the highest number of DEGs (2756). The consensus DEGs can be explored in our online database, accessible at https://covidatlas.sysbio.tools/.

**Figure 2:**
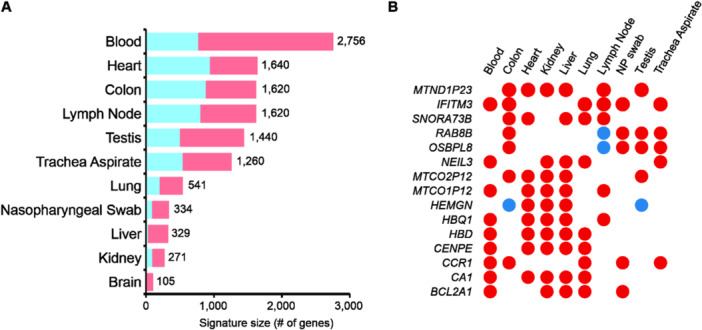
Discovery of key genes linked to severe COVID‐19. (A) Number of consensus genes identified in bulk RNA‐seq data, categorized by tissue. Upregulated genes are represented in pink, while downregulated genes are depicted in cyan. (B) Hub genes identified through network analysis, where nodes represent consensus genes connected with tissues. Nodes with a degree ≥ 5 are highlighted. Node color indicates the median log2 fold change for each gene in each tissue, with red representing upregulation and blue representing downregulation.

In contrast, despite being a primary target of COVID‐19 pathology, the lung presented only 541 DEGs. Organs such as the heart, colon, lymph nodes, and testis showed more DEGs than the lung, nasopharyngeal swab, liver, kidney, and brain (Figure 2A).

Further investigation through network analysis (Figure S5) identified 15 hub genes—defined as having a node degree ≥ 5—within the Gene–Tissue Network. These include IFITM3, MTND1P23, CA1, CCR1, CENPE, HBD, HBQ1, MTCO1P12, NEIL3, BCL2A1, OSBPL8, RAB8B, SNORA73B, MTCO2P12, and HEMGN. Collectively, they are present in 5 of the 11 tissues analyzed (~45%), suggesting a potential role in the systemic impact of severe disease. Among them, IFITM3 stood out, being notably upregulated in 6 of the 11 tissues (~54%), including blood, nasopharyngeal swabs, lung, tracheal aspirate, lymph node, and colon. This gene is known to play a role in the antiviral response to SARS‐CoV‐2, with specific genetic variants linked to increased risk of severe disease [22, 23]. A detailed view of the differential expression of these hub genes is shown in Figure 2B. An interactive version of the network is available at https://degs.covidatlas.sysbio.tools/.

### 2.3 Consensus Functional Alterations in Severe COVID‐19

**Figure 3:**
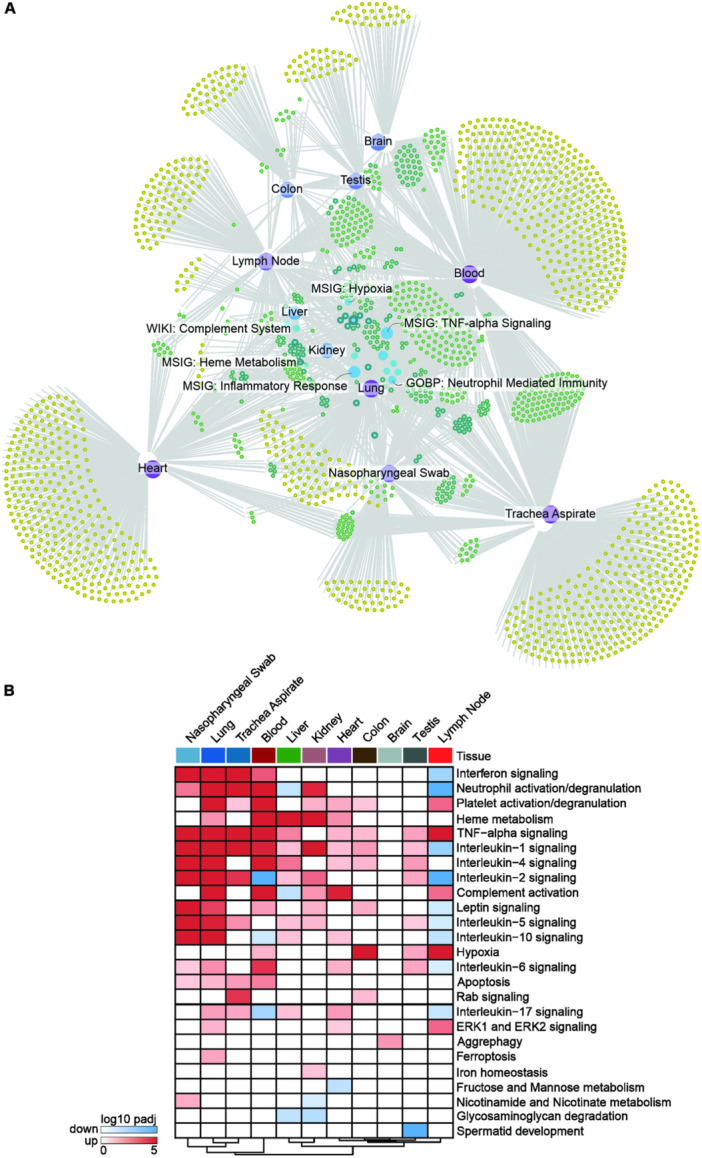
Unveiling central and tissue‐specific pathways linked to severe COVID‐19. (A) Exploration of hub pathways through network analysis, where nodes represent enriched pathways associated with consensus genes connected with tissues in bulk RNA‐seq data. Nodes with a degree ≥ 5 are highlighted, shedding light on central pathways potentially orchestrating the molecular response in severe COVID‐19 across diverse tissues. An interactive version of the network can be found at https://pathways.covidatlas.sysbio.tools/. (B) Enrichment analysis results illustrate enriched pathways using consensus gene sets identified as common and tissue‐specific in severe COVID‐19. Pathways are color‐coded, with blue indicating downregulation and red indicating upregulation in bulk RNA‐seq data.

We identified 1992 enriched pathways across various tissues for consensus DEGs using bulk RNA‐seq data (Supporting Information S4: Table 3). Network analysis of these pathways revealed key biological processes, including inflammatory response, TNF‐alpha signaling via NF‐κB, and neutrophil‐mediated immunity, which were consistently perturbed across most tissues examined (Figure 3A).

However, some inconsistencies emerged. For example, the MSigDB Hallmark 2020 Inflammatory Response pathway was upregulated in seven tissues but downregulated in one, whereas the same pathway, according to GO Biological Process 2021, was upregulated in only four tissues. These discrepancies highlight the variability among gene set annotation databases and underscore the need for a more refined analytical approach.

To address this complexity, we implemented a customized strategy (Section 5.7). This approach reaffirmed the significance of inflammatory response, TNF‐α signaling, and neutrophil‐mediated immunity, while also uncovering tissue‐specific processes. Notable examples include Rab signaling in the tracheal aspirate and colon, aggrephagy in the brain, and ferroptosis in the lung (Figure 3B and Supporting Information S5: Table 4). These findings illustrate the diverse molecular landscape of severe COVID‐19 and emphasize the importance of targeted therapeutic interventions that consider tissue‐specific variations.

### 2.4 Cell‐Specific Immune Dysregulation and Potential Critical Genes in Severe COVID‐19

Next, we explored enriched pathways at the single‐cell level, focusing on those previously identified through bulk RNA‐seq. Interferon signaling was broadly upregulated across several tissues. However, notable exceptions were observed in specific cell types—such as CD8 T cells in nasopharyngeal swabs and vascular endothelial cells in the lung—where this pathway was downregulated. These findings underscore the cell‐type‐specific modulation of interferon signaling in severe COVID‐19, particularly in T cells and endothelial cells.

**Figure 4:**
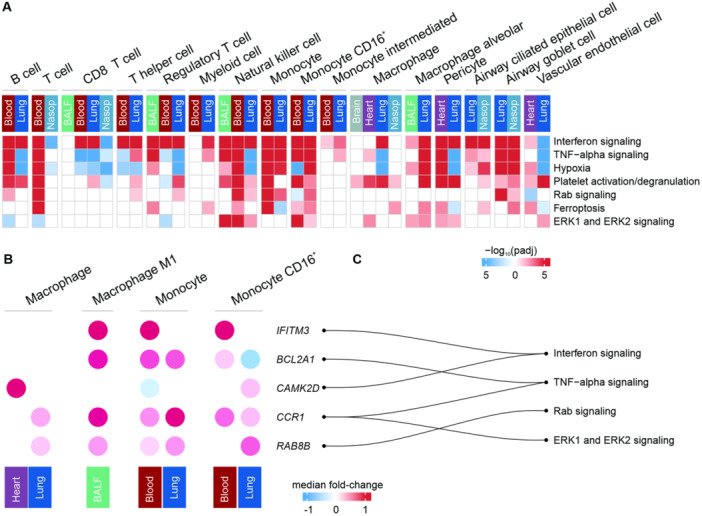
Unraveling differentially expressed genes and pathways in severe COVID‐19 across cell types. (A) Enriched Pathways in scRNAseq Data. Identification of enriched pathways in bulk RNA‐seq data, specifically focusing on their presence in scRNAseq data. Enrichment scores are represented in blue for downregulated and red for upregulated pathways. Top annotations specify the associated tissue and cell types, providing insights into the dysregulated pathways across different cell populations. (B) Differential expression of hub genes across cell types in scRNAseq data. Exploration of the differential expression of hub genes, previously identified in bulk RNA‐seq data, now extended to scRNAseq data within monocytes and macrophages across blood, lung, heart, and bronchoalveolar lavage fluid (BALF). The median average log fold change (avglogFC) for upregulated genes is depicted in red, while downregulated genes are shown in blue. (C) Signaling pathways connected to critical genes in monocytes and macrophages. Investigation of signaling pathways connected to identified essential DEGs in monocytes, monocytes CD16, coarse macrophages, and macrophages M1. This section focuses on genes overlapping in the enrichment analysis results and their associated signaling pathways, providing a more detailed understanding of molecular alterations in specific cell types.

Further analysis revealed distinct enrichment patterns within subsets of immune cells. For instance, monocytes, intermediate monocytes, and CD16‐positive monocytes exhibited contrasting expression profiles. Ferroptosis, a form of programmed cell death, was upregulated in the lung but downregulated in the nasopharyngeal swab. In contrast, Rab signaling, which is essential for intracellular transport, showed an inverse pattern in CD16‐positive monocytes compared to the lung (Figure 4A). These observations highlight the complexity of cellular responses across different tissues in severe COVID‐19.

We also examined the expression of hub genes in monocytes and macrophages across tissues. Genes such as IFITM3, BCL2A1, CAMK2D, CCR1, and RAB8B showed differential expression in blood, lung, bronchoalveolar lavage fluid (BALF), and heart tissues (Figure 4B). Their consistent involvement supports their potential role in the pathogenesis of severe COVID‐19, particularly through pathways such as interferon and TNF‐α signaling (Figure 4C). A comprehensive list of DEGs, enriched pathways, and biological processes from the scRNA‐seq analysis is provided in Supporting Information S6–S8: Tables [Link], [Link], [Link].

### 2.5 RAB8B: A Key Modulator in COVID‐19 Pathophysiology and Viral Replication

Our analysis suggests a differential expression of RAB8B across various tissues and cell types, marking it as a gene of interest in COVID‐19. Notably, RAB8B showed significant upregulation in CD16‐positive monocytes and CD16hi NK cells within lung and blood tissues, respectively. Additionally, inflammatory macrophages in BALF and lung tissues exhibited increased RAB8B expression (Figure 6A,B). These patterns suggest a potential role of RAB8B in severe COVID‐19 pathophysiology.

Candidates were selected from our atlas to compare with a CRISPR meta‐analysis [24], revealing potential targets for investigating their involvement in SARS‐CoV‐2 replication (Supporting Information S9: Table 8). Among the genes identified in the CRISPR screening were several RAB genes, including RAB21, RAB2A, RAB32, RAB3A, RAB3B, RAB6B, and RAB7A. RABs are recognized for their association with the viral replication of enveloped viruses that infect humans [25]. Interestingly, while RAB8B was identified in our hub genes, it was not present in the CRISPR screening. Therefore, we decided to validate its role in SARS‐CoV‐2 replication.

Experimental data with Jurkat cells indicate that RAB8b recruits the (v)‐SNARE protein VAMP‐3 [26], a critical component involved in endosome fusion with the immune synapse membrane. To investigate the role of RAB8b in VAMP‐3 recruitment, we employed atomistic (AA) and coarse‐grained (CG) molecular dynamics simulations. Using AA simulations, we modeled RAB8b in aqueous solution and VAMP‐3 embedded in a POPC membrane (Supporting Information S1: Figure 7A). Analysis of the backbone root mean square deviation (RMSD) and root mean square fluctuation (RMSF) revealed that, apart from the N‐ and C‐terminal regions, the overall protein structures of RAB8b and VAMP‐3 remained stable in solution and within the membrane, respectively (Supporting Information S1: Figure 7B).

**Figure 5:**
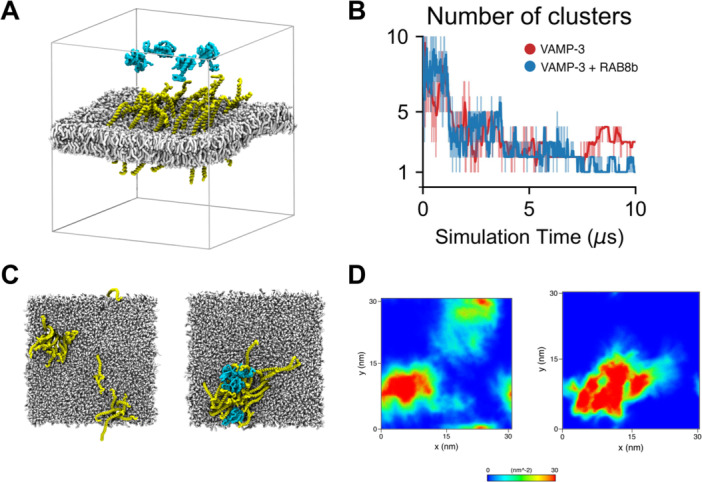
RAB8b modulates VAMP‐3 distribution. (A) Initial structure of the RAB8b and VAMP‐3 system in the coarse‐grained (CG) POPC membrane simulation. RAB8b is depicted in cyan, VAMP‐3 in yellow, and POPC lipids in silver. (B) Average number of VAMP‐3 clusters observed in the control simulation (red) and the RAB8b‐containing simulation (blue). (C) Top‐view of the final snapshots of the simulations showing the spatial distribution of VAMP‐3 in the control and RAB8b‐containing systems. (D) Two‐dimensional number density maps of VAMP‐3 in the control and RAB8b‐containing simulations, averaged over the last 500 ns of the simulations.

With CG simulations, we simulated four RAB8b monomers with 25 VAMP‐3 proteins embedded in a POPC membrane for 10 µs (Figure 5A), and compared these results to a control simulation with 25 VAMP‐3 proteins in a POPC membrane (Supporting Information S1: Figure 7C, right side). In the control simulation, VAMP‐3 exhibited self‐clustering behavior, with an average of 2.6 ± 0.7 clusters observed during the last 5 µs (Figure 5B). In contrast, in the presence of RAB8b, the average number of VAMP‐3 clusters was reduced to 1.7 ± 0.7 (Figure 5B), suggesting that RAB8b facilitates VAMP‐3 clustering in the membrane. This observation was further validated by 2D density plots, which corroborated the clustering behavior (Figure 5C,D).

**Figure 6:**
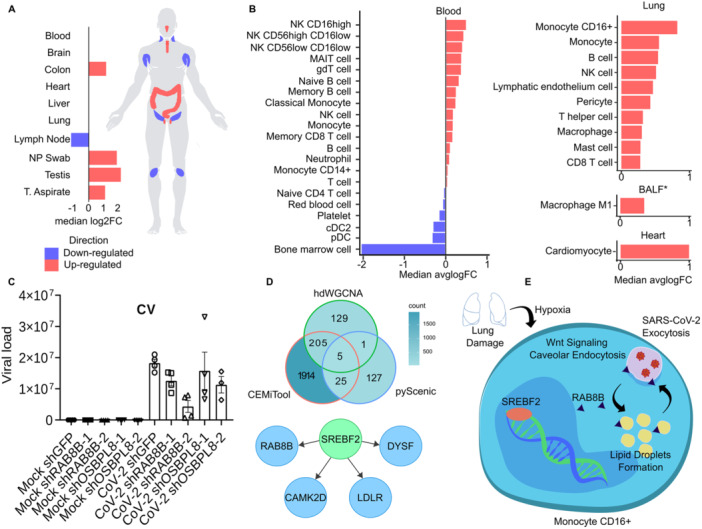
Differential expression and molecular mechanisms of RAB8B associated with severe COVID‐19. (A) Schematic representation and bar chart illustrating the median log2 fold change (log2FC) of RAB8B across various tissues in bulk RNA‐seq data obtained from severe COVID‐19 cases. (B) The bar chart presents the median avglogFC of RAB8B in different cell types, as revealed by scRNA‐seq data in severe COVID‐19. (C) In vitro silencing experiment of RAB8B and OSBPL8 in CaCo‐2 cells infected with SARS‐CoV‐2, compared to mock‐infected cells. Results demonstrate a significant reduction in viral load, specifically for RAB8B. (D) The Venn diagram depicts the common and unique genes coexpressed with RAB8B using three methods (Pseudobulk + CEMiTool, hdWGCNA, and pyScenic). Below is a regulatory network indicating SREBF2 as a potential transcription factor regulating the expression of RAB8B, CAMK2D, DYSF, and LDLR. (E) Schematic representation of our postulated hypothesis elucidating the molecular mechanism associated with RAB8B upregulation and SARS‐CoV‐2 exocytosis in severe COVID‐19.

To further understand the molecular mechanisms underlying RAB8B, we employed three correlation analysis methods (hdWGCNA, Pseudobulk + CEMiTool, and pyScenic), identifying four genes (CAMK2D, DYSF, LDLR, and SREBF2) consistently associated with RAB8B. This multi‐methodological approach underscored the robustness of our findings and suggested a shared biological significance among these genes. Additionally, pyScenic implicated the transcription factor SREBF2 as a potential regulator of genes correlated with RAB8B, offering insights into the gene's regulatory network in severe COVID‐19 (Figure 6D and Supporting Information S11: Table 10).

Prompted by the similar RAB8B and OSBPL8 differential expression profiles, we explored their roles in SARS‐CoV‐2 replication. Utilizing CaCo‐2 cells and a lentiviral shRNA approach for gene silencing. Compared to the shGFP control, knockdown of Rab8b‐1 resulted in 30% reduction in SARS‐CoV‐2 infection (*p* = 0.0302). In addition, knockdown of Rab8b‐2 caused a pronounced 76% decrease in viral infection (*p* < 0.001), indicating a reproducible and strong functional role for Rab8b to promote SARS‐CoV‐2 infection (Figure 6C and Supporting Information S10: Table 9).

### 2.6 Interactive Online Database

**Figure 7:**
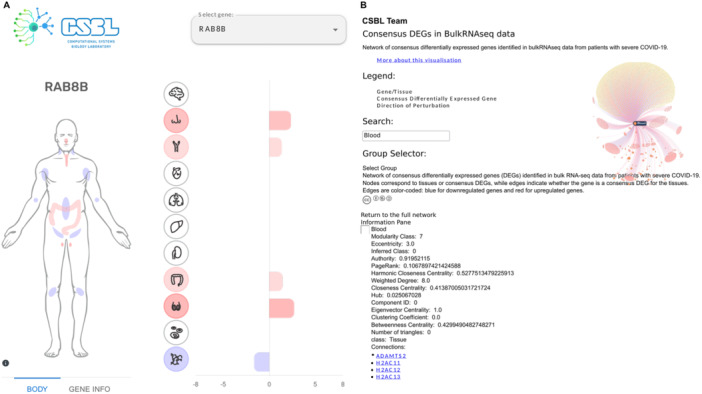
Interactive online database for transcriptome of severe COVID‐19. This figure presents screenshots of the interactive features available in an online database for exploring transcriptomic data related to severe COVID‐19. (A) The first screenshot displays an interactive bar chart depicting the median log2 fold change (log2FC) of the gene RAB8B across various tissues in bulk RNA‐seq data obtained from severe COVID‐19 patients (https://covidatlas.sysbio.tools/). (B) The second screenshot showcases the exploration of consensus gene signatures through an interactive network visualization tool. Nodes within the network represent consensus genes, and connections indicate associations with specific tissues in bulk RNA‐seq data from severe COVID‐19 patients. Additionally, network metrics are displayed, offering users a deeper understanding of gene–tissue relationships and network topology (https://pathways.covidatlas.sysbio.tools/).

To provide interactive and user‐friendly access to our consensus signatures and enriched signaling pathways, we developed an online database. This platform enables researchers to search for genes of interest and visualize their consensus differential expression status across various tissues, including their related enriched pathways. For instance, a researcher could query “RAB8B” at https://covidatlas.sysbio.tools/ to promptly assess its differential expression, observing that it is upregulated in the nasopharynx, trachea, colon, and testis, and downregulated in the lymph node (Figure 7A). The database also offers interactive network visualizations for consensus DEGs, pathways, and tissues, allowing users to explore gene‐tissue or pathways‐tissue associations. If a user searches “Blood” at https://pathways.covidatlas.sysbio.tools/, they are going to find some network metrics, such as modularity class, clustering coefficient, eccentricity, among others. They are also going to find a list of genes that are differentially expressed, such as ADAMTS2, H2AC11, H2AC12, among others (Figure 7B). This resource provides a comprehensive and intuitive interface for delving into the molecular dynamics of severe COVID‐19 across different tissues.

## 3 Discussion

Our integrative transcriptomic analysis, encompassing 39 studies with 2493 samples and over 2 million cells, culminated in a comprehensive multiorgan atlas of severe COVID‐19. This atlas delineates robust consensus gene signatures across 11 tissues and underscores the systemic nature of the disease. Central to these signatures are key molecular hubs—IFITM3, BCL2A1, CAMK2D, CCR1, and RAB8B—which converge on critical signaling pathways, including interferon, TNF‐α, ERK1/ERK2, and Rab signaling. Notably, RAB8B has been implicated in inhibiting SARS‐CoV‐2 replication in vitro, highlighting its therapeutic potential.

Our network‐based analyses revealed consistent dysregulation of inflammatory pathways such as neutrophil degranulation and TNF‐α signaling across multiple organs [27, 28, 29], in line with the hyperinflammatory and prothrombotic milieu characteristic of severe COVID‐19 [30]. Additionally, upregulation of platelet degranulation and complement activation pathways in key tissues suggests their contributory role in driving immunothrombosis and exacerbating tissue injury [31].

Tissue‐specific dysregulations also emerged. In nasopharyngeal swabs, we observed upregulation of the nicotinamide salvaging pathway, which is crucial for maintaining cellular NAD+ pools involved in energy metabolism, gene regulation, stress responses, and DNA repair [32]. In viral infections, nicotinamide supports PARP‐mediated ADP‐ribosylation, a mechanism essential for innate immunity [33]. SARS‐CoV‐2 appears to counteract this by hydrolyzing ADP‐ribose via its Nsp3 macrodomain, subverting host defenses [34]. This disruption has been hypothesized to contribute to pellagra‐like symptoms observed in some COVID‐19 patients [35], though systemic implications remain to be elucidated.

In kidney and liver tissues, we noted downregulation of glycosaminoglycan (GAG) degradation, a pathway implicated in mucopolysaccharidoses. These lysosomal storage disorders are characterized by renal and hepatic pathology resulting from GAG accumulation [36]. Within affected individuals, manifestations such as nephrotic syndrome, renal failure, hepatomegaly, and cirrhosis are attributed to the accumulation of undegraded GAGs within the lysosomes of kidney and liver cells, precipitating cellular dysfunction, inflammation, oxidative stress, and tissue impairment [36]. Although our findings mirror some manifestations observed in severe COVID‐19, no direct causal link has yet been established, warranting further investigation.

In brain tissue, aggrephagy (GO:0035973)—a selective autophagic process targeting protein aggregates [37]—was significantly upregulated. Viral infections, including SARS‐CoV‐2, may exploit this mechanism to enhance replication. Emerging evidence suggests that viral infections, including SARS‐CoV‐2, can activate aggrephagy to enhance viral replication [38]. Specifically, the SARS‐CoV‐2 ORF7a protein has been shown to activate LC3‐II and suppress SNAP29, impeding autophagosome–lysosome fusion and promoting viral persistence [39]. These findings suggest a possible mechanism contributing to neurological manifestations in COVID‐19, although further validation is needed.

Cross‐validation with scRNA‐seq data confirmed a broad upregulation of interferon signaling at the single‐cell level in most tissues, with exceptions in T cells and CD8+ T cells from nasopharyngeal swabs and in lung endothelial cells. This downregulation may impair local antiviral responses and facilitate systemic viral spread. IFITM3, one of the most consistently upregulated genes, is a known mediator of viral restriction and susceptibility to SARS‐CoV‐2 [23].

The depletion of T cells—especially CD8+ T cells—is a hallmark of severe disease, potentially reflecting delayed or inadequate activation [40]. Impaired interferon signaling at viral entry sites like the nasopharynx may compromise early containment, allowing progression to systemic infection. Moreover, downregulation of interferon pathways in pulmonary endothelial cells may contribute to endothelial dysfunction and thrombus formation [41, 42].

We also observed widespread upregulation of platelet activation signatures, particularly in macrophages from brain and heart tissue. This supports recent findings on increased platelet‐monocyte aggregates in severe COVID‐19 and highlights the growing recognition of platelet–macrophage crosstalk in disease pathogenesis [43].

Crosstalk between hypoxia and TNF‐α signaling in monocytes/macrophages was also evident. Hypoxia has been shown to amplify TNF‐α production in macrophages [44, 45], and TNF‐α can upregulate CA1, a consensus DEG identified in our analysis [46]. These interactions emphasize the central role of monocytes/macrophages in orchestrating the inflammatory response. CCR1, another hub gene, is linked to inflammatory monocyte recruitment [47], while BCL2A1, also upregulated, is known to enhance monocyte/macrophage survival under pro‐inflammatory conditions [48].

Finally, we identified NEIL3 and CAMK2D as novel hub genes in both bulk and single‐cell data sets across multiple tissues. CAMK2D, a calcium/calmodulin‐dependent kinase activated by ROS, regulates Ca^2+^ signaling and inflammatory responses [49]. It has been implicated in inflammasome activation and macrophage recruitment in murine models of cardiac injury [50]. NEIL3, a DNA glycosylase, plays a key role in oxidative DNA damage repair [51]. While their specific roles in COVID‐19 remain underexplored, their consistent upregulation suggests potential involvement in tissue‐specific responses to oxidative and inflammatory stress.

RAB8B, a small GTPase involved in membrane trafficking, Wnt signaling, and lipid droplet formation [52, 53, 54], emerges as a potential proviral factor in SARS‐CoV‐2 infection. Our in vitro silencing of RAB8B significantly reduced viral replication, while molecular dynamics simulations showed it facilitates VAMP‐3 clustering on membranes—highlighting its role in vesicle trafficking and membrane fusion. Notably, RAB8B has also been implicated in the exocytosis of West Nile virus, suggesting a conserved mechanism [55].

We hypothesize that SARS‐CoV‐2–induced lung damage creates a hypoxic environment that activates SREBF2, a transcription factor linked to cholesterol biosynthesis [56]. This, in turn, may upregulate RAB8B, promoting endosomal recycling and lipid droplet formation—processes hijacked by the virus to enhance replication. Supporting this, lipid droplet accumulation has been observed in SARS‐CoV‐2–infected monocytes [57], and increased intracellular lipids are essential for viral replicon formation [58].

Together, these findings suggest a hypoxia‐induced SREBF2–RAB8B axis that promotes SARS‐CoV‐2 replication by driving metabolic and vesicular remodeling (Figure 5E). Pharmacological inhibition of SREBP pathways has been shown to reduce inflammasome activation and cell death, underscoring its potential as a therapeutic target [59]. Additionally, targeting RAB GTPases to disrupt extracellular vesicle trafficking has been proposed as an antiviral strategy [60]. Such interventions may help lower viral load and alleviate severe COVID‐19 symptoms. However, challenges related to specificity, delivery, and off‐target effects must be addressed.

While our study offers a comprehensive multiorgan transcriptome atlas of severe COVID‐19, it is important to acknowledge certain limitations. First, our analysis relies on the integration of publicly available data sets, which inherently introduces cross‐study variability and potential batch effects despite our rigorous normalization and consensus‐building approaches. While we employed robust statistical methods to mitigate these issues, complete elimination of such variability is challenging. Second, the generalizability of our in vitro experimental findings, particularly concerning RAB8B's role in SARS‐CoV‐2 replication, requires further validation in more complex in vivo models that better recapitulate the human physiological environment. Third, our atlas primarily focuses on the molecular landscape of severe COVID‐19, meaning the identified signatures and pathways may not fully represent the spectrum of molecular changes observed in milder forms of the disease. Finally, as previously noted, the absence of comprehensive patient metadata (e.g., age, sex, comorbidities) across all original studies limits our ability to fully explore demographic‐specific molecular responses and potential confounding factors, emphasizing the critical necessity for future research to prioritize the availability of inclusive data. These limitations notwithstanding, our study provides a valuable foundation for understanding the systemic molecular pathology of severe COVID‐19 and serves as a robust resource for future investigations.

## 4 Conclusions

Our study provided reliable consensus gene signatures across different tissues. Furthermore, our findings provide valuable insights, underscoring the intricate nature of COVID‐19's molecular mechanisms and the imperative for further research. A significant discovery from our study is the association of RAB8B with disease severity and its impact on SARS‐CoV‐2 replication in vitro. Such validation could represent a substantial leap forward in developing COVID‐19 treatment strategies, underscoring the importance of continued research in this field and highlighting the potential for these findings to translate into improved diagnostics, targeted therapies, and more effective patient management in clinical settings.

Building upon this comprehensive atlas, several promising avenues for future research emerge. First, the in vivo validation of our key findings, particularly the mechanistic role of RAB8B in SARS‐CoV‐2 infection and its proposed axis with SREBF2 and lipid metabolism, is crucial. This would involve studies in relevant animal models to confirm the observed effects in a physiological context. Second, the identification of RAB8B as a potential modulator of viral replication opens the door for drug screening efforts aimed at identifying small molecules or compounds that can inhibit RAB8B activity or modulate its associated pathways. Such efforts could lead to the development of novel antiviral therapeutics. Third, our integrated multiorgan approach can be extended to investigate the molecular underpinnings of post‐acute COVID‐19 (Long COVID), providing insights into the persistent symptoms and long‐term sequelae of the disease. Finally, applying this framework to analyze emerging SARS‐CoV‐2 variants could help elucidate how viral evolution impacts host responses and disease severity, further enhancing our understanding of this complex pathogen.

## 5 Materials and Methods

### 5.1 Study Selection and Transcriptome Data Collection

A comprehensive literature review was conducted to identify studies providing transcriptomic data (bulk RNA‐seq or scRNA‐seq) from human samples related to COVID‐19. Inclusion criteria encompassed studies published between December 2019 and July 2021, featuring samples from relevant tissues (lung, blood, brain, heart, kidney), clearly defined clinical severity (particularly severe or critical COVID‐19), and available lists of DEGs or raw data suitable for reanalysis. Sufficient metadata, including tissue type, disease status, and experimental conditions, was also required.

Exclusion criteria included the absence of patient‐level metadata for disease severity categorization, insufficient sequencing depth or quality, the exclusive use of in vitro or nonhuman models, lack of control samples, analysis limited to a single cell type, absence of publicly deposited raw data, duplicate publications, or a focus on noncoding RNAs. After applying these criteria, 39 studies were selected for further analysis. Lists of DEGs from COVID‐19 patients and control cases were compiled, adhering to the significance cutoff values provided by the original authors. Alongside DEGs, relevant metadata such as comparisons, group descriptions, sample sizes, tissue and cell types, and statistical tests were extracted. Statistical cutoff values for log2FC, *p* values, and adjusted *p* values were recorded. Sequencing type (Bulk‐RNA or scRNA‐seq) and study attributes (titles, access links, codes, and data repository information) were also documented.

### 5.2 Reanalysis of Bulk RNA‐Seq Studies

#### 5.2.1 Reanalysis of Raw Data

For studies lacking lists of DEGs and raw count matrices, raw RNA sequencing data were retrieved from the Gene Expression Omnibus. FASTQ files were downloaded using SRA Toolkit v2.10.9 [61]. Quality control involved filtering sequences with Phred scores below 30, sequences shorter than 36 bases, and adapter sequences using FASTP v0.23.2 [62]. Reads passing quality control were aligned and mapped to the Homo sapiens reference genome (Homo_sapiens.GRCh38.dna.primary_assembly.fa) and gene model (Homo_sapiens.GRCh38.104.gtf) using STAR v2.7.9a default parameters [63]. The integrity and quality of reads were assessed before and after preprocessing using FastQC v0.11.9, and a summary of quality metrics was compiled using MultiQC v1.12 [64, 65].

#### 5.2.2 Differential Expression Analysis

For studies without provided DEG tables, differential expression analysis was performed using raw count tables from re‐sequencing or original studies. This involved removing genes with zero counts, identifying outliers using Cook's distances and MDP scores, and employing DESeq2 v1.40.2 [66, 67]. A standard statistical cutoff of an adjusted *p* value (padj) < 0.01 and an absolute log2FC > 1 was applied. An exception was made for study number 62 (brain tissue), where a padj < 0.05 was used without a log2FC threshold due to the low number of DEGs detected with more stringent criteria. Gene identifiers were converted from ENSEMBL IDs to Gene Symbols using BioMart v2.58.0, and ggplot2 v3.4.4 was used for visualization [68, 69].

#### 5.2.3 Reanalysis of scRNA‐Seq Studies

In studies where lists of DEGs were not provided by the authors, available matrices from the 10× Genomics platform (matrix.mtx, genes.tsv/features.tsv, and barcodes.tsv) were utilized. Subsequent analyses followed the standard Seurat v4.3.0.1 workflow [70, 71]. Quality control involved excluding cells with fewer than 200 detected genes or more than 10% mitochondrial gene content. Data were log‐normalized using NormalizeData() with default parameters. Highly variable features were identified using FindVariableFeatures(), and the top 2000 highly variable genes were used to scale data with ScaleData() and perform dimensionality reduction via principal component analysis. Clustering was performed through neighborhood graph construction and clustering using FindNeighbors() and FindClusters() functions, applying a resolution of 0.5 and using the first 20 principal components. UMAP was computed using RunUMAP() for visualization, also with the first 20 dimensions. Cell type annotation was performed manually using marker genes described in the original publications. Differential expression analysis was conducted using the FindMarkers() function, comparing COVID‐19 samples to controls for each identified cell type, with a Wilcoxon statistical test and a log2FC threshold of 0.25. No batch correction was required as each data set was processed separately under the same experimental conditions.

### 5.3 Identification of Consensus Gene Signatures in Bulk RNA‐Seq Data

Lists of DEGs were categorized by sequencing method, tissue, and cell type. All lists of DEGs were filtered for statistical significance. For author‐provided lists of DEGs, original thresholds were adhered to. For reanalyzed data sets, a standardized statistical cutoff of padj < 0.01 and |log2FC| > 1 was applied, with the exception of data set #62 (brain tissue), where padj < 0.05 was used without a log2FC cutoff.

For groups with multiple bulk RNA‐seq lists of DEGs, consensus DEGs were identified using a vote‐counting approach, retaining genes with an absolute sum of expression‐direction votes of two or more. Median log2FC values were calculated, and combined *p* values were determined using Fisher's method to assess statistical significance across multiple studies.

In groups with a single lists of DEGs, DEGs were classified as up‐regulated or down‐regulated and ranked based on a score calculated as score = |log2FC| × −log2(padj). The top 20 DEGs by score underwent enrichment analysis using Reactome and KEGG databases, employing the “fora” function from FGSEA v1.26.0 [72, 73]. Pathways with 15–500 genes were targeted, and biologically relevant pathways were identified as those with padj < 0.05 and at least 5 gene overlaps. The number of DEGs analyzed increased iteratively by 20, up to 1000, to explore gene sets and assess pathway enrichment stability. All enrichment results were compiled to determine the total number of enriched pathways and median size for each tissue and expression direction, determining consensus DEGs based on the highest total number of enriched pathways and the smallest median size.

### 5.4 Testing Robustness of Consensus Signatures

The representativeness of identified consensus DEGs compared to original signatures was evaluated, hypothesizing that robust consensus signatures would show a higher degree of gene overlap within stratified categories (tissue type, sequencing method, clinical condition). Enrichment scores (−log10 *p* value) for shared genes and enriched pathways between each list of DEGs were calculated using Fisher's exact test, implemented with the “fora” function from clusterProfiler v4.9.0.002 [74]. A symmetric matrix based on these scores was constructed, followed by hierarchical cluster analysis using Pearson's correlation for distance measurement and the average agglomeration method. Results were visually displayed using ComplexHeatmap v2.16.0 [75]. Concordance between original gene signatures and consensus DEGs was assessed by comparing hierarchical grouping outcomes.

### 5.5 Functional Enrichment Analysis

Overrepresentation analysis of consensus DEGs from bulk RNA‐seq and individual gene signatures from scRNA‐seq studies (separately for upregulated and downregulated genes) was performed using Fisher's exact test. Annotation data sets included “Reactome 2022,” “BioCarta 2016,” “BioPlanet 2019,” “GO Biological Process 2021,” “GO Cellular Component 2021,” “GO Molecular Function 2021,” “MSigDB Hallmark 2020,” “Panther 2016,” and “WikiPathway 2021 Human” [76, 77, 78, 79, 80, 81, 82]. All human protein‐coding genes from the ENSEMBL database served as the reference universe. Enrichment analysis focused on pathways with 15–500 genes, and pathways with an adjusted *p* value below 0.05 were considered statistically significant. These analyses were conducted using FGSEA v1.26.0 and hypeR v2.0.1 [72, 83, 84].

### 5.6 Integrative Analysis of Consensus Signatures and Enriched Pathways in Bulk RNA‐Seq

Network analyses were performed on consensus DEGs and functional enrichment data from bulk RNA‐seq studies, resulting in two network types: Gene–Tissue Network: Nodes represent consensus DEGs and tissues; an undirected, unweighted edge connects a consensus DEG to a tissue if the gene was identified as a consensus DEG in that tissue. Pathway–Tissue Network: Nodes represent enriched pathways and tissues; edges connect a tissue to a given pathway if it was found as significantly enriched (FDR ≤ 0.05) in that tissue. Edge weights correspond to −log10(FDR), but all network metrics were computed on the unweighted graph.

In both networks, layouts were generated using the Fruchterman‐Reingold algorithm (area = 800, gravity = 0.5, velocity = 1.0), node size was mapped to degree, and node color to modularity class. Fundamental network properties such as density, diameter, degree, degree distribution, clustering coefficient, and centrality measures (betweenness, closeness, and eigenvector centrality) were calculated. In the Gene–Tissue Network, a threshold was set for node degree, identifying genes with a degree ≥ 5 as the most representative across tissues. Gephi v0.10.1 software was utilized for network construction and analysis [85].

### 5.7 Functional Enrichment Analysis of Selected Biological Processes Using Consensus Gene Sets

A tailored approach was implemented to minimize redundancy in functional enrichment results. A custom collection of “Consensus Gene Sets” was constructed by extracting genes from curated gene sets whose titles matched predefined keywords corresponding to biological processes relevant to the study (e.g., immune‐related, cellular stress responses, metabolic, signaling). Pre‐annotated gene sets containing protein‐coding genes from BioCarta 2016, BioPlanet 2019, GO Biological Process 2021, GO Cellular Component 2021, GO Molecular Function 2021, MSigDB Hallmark 2020, Panther 2016, and WikiPathway_2021_Human were loaded. A case‐insensitive search for matching terms within gene set names was performed using regular expressions. All gene sets with matching names were selected, and associated genes were extracted and merged, retaining only unique gene symbols per process. This strategy defined gene modules representing each biological process, minimizing redundancy. The resulting gene sets were used in downstream functional enrichment analysis applied to both bulk RNA‐seq and scRNA‐seq data using this customized annotation data set, employing Fisher's exact test. Protein‐coding genes from the ENSEMBL database were used as the reference universe. Upregulated and downregulated genes were analyzed separately. An adjusted *p* value threshold of ≤ 0.05 was used for statistical significance. These analyses were conducted using FGSEA v1.26.0 and hypeR v2.0.1 [72, 83, 84].

### 5.8 Integrative Analysis of scRNA‐Seq Data

To address heterogeneity in cell type annotation, a hierarchical ontological framework for cell classification was implemented within each tissue, based on the Cell Ontology from Wikidata and manual curation of author‐provided labels [86]. For each scRNA‐seq study, pathway enrichment analyses were performed separately for upregulated and downregulated genes as described in Supplementary Methods: S12 (Section 6), using overrepresentation analysis with an adjusted *p* value cutoff of < 0.05 for significance and FGSEA v1.26.0 [72]. The enrichment direction was defined according to the input gene list. If the same pathway was significantly enriched (padj < 0.05) for both upregulated and downregulated genes within a cell type, the direction was assigned based on the lowest adjusted *p* value.

To determine the consensus direction of enrichment across cell types, a vote‐counting strategy was applied: +1 for upregulated enrichment and −1 for downregulated. A consensus direction was assigned when the sum of votes was ≥ 2 or ≤ −2. For consistent cases, adjusted *p* values from studies with the same direction were combined using Fisher's method. Results from cell subsets were not aggregated to infer consensus enrichment for broader cell type categories. Finally, shared cell types or subsets across tissues were identified and used to compare enrichment scores of the same cell type across different tissues.

### 5.9 Molecular Dynamics Simulations

#### 5.9.1 All‐Atom Simulations Set‐ups and Analysis

All‐atom (AA) simulations were performed using the CHARMM36m [87] force field for proteins and lipids, along with the TIP3P [88] water model. Simulations were run with a time‐step of 2 fs and all bonds were constrained using the LINCS algorithm [89]. Neighbor‐searching was accomplished using the Verlet cut‐off scheme at every 50 fs. Short‐range electrostatic and van der Waals interactions were computed using a 1.2 nm cutoff, using a potential‐switch function starting at 1.0 nm. Long‐range electrostatic interactions were treated using the Particle Mesh Ewald method [90]. The system temperature was maintained at 310 K using the velocity‐rescaling thermostat [91] with a 0.1 ps coupling constant. Semi‐isotropic pressure was applied with the Parrinello–Rahman barostat [92] at 1 bar, using a 1.0 ps coupling constant. Energy minimization step was performed using the steepest descent method, followed by NVT and NPT steps. For each system, three independent simulations were conducted for 300 ns. For the structural analyses, RMSD and RMSF were calculated using gmx rmsd and gmx rms commands, respectively, after fitting the proteins to their initial backbone structures using the gmx trjconv command.

#### 5.9.2 RAB8b in Water

The RAB8b structure was obtained from the AlphaFold Protein Structure Database [93] (UniProt Q92930). The protein was inserted into a 10 × 10 × 10 nm simulation box and solvated with TIP3P water molecules. Counter‐ions (K+ and Cl−) were added to neutralize the system, and position restraints on the protein backbone were applied during minimization, NVT, and NPT equilibration steps.

#### 5.9.3 VAMP‐3 in a POPC Bilayer

The VAMP‐3 structure was also obtained from the AlphaFold Protein Structure Database [34] (UniProt Q15836). The protein/membrane system was built using CHARMM‐GUI MembraneBuilder [94, 95, 96]. The bilayer contained 72 POPC lipids per leaflet, with 1 VAMP‐3 oriented perpendicularly to the bilayer. The system was solvated with 6.5 nm thick water slabs on both sides of the membrane and neutralized with K+ and Cl− counter‐ions. Position restraints were applied on the protein backbone during minimization, NVT, and NPT equilibration steps.

#### 5.9.4 Coarse‐Graining Simulations Set‐ups and Analysis

Coarse‐graining (CG) simulations were performed using the Martini3 force field [97]. A time step of 20 fs was used, with neighbor searching updated every 20 steps using the Verlet algorithm. van der Waals interactions we computed with a 1.1 nm cutoff, and Coulomb interactions were treated using the reaction‐field method with a cutoff of 1.1 nm with a dielectric constant of 15. Semi‐isotropic pressure coupling was applied using the C‐rescale barostat [98] at 1 bar, with a coupling constant of 4.0 ps^−1^, and compressibility of 3 × 10^−4^ bar^−1^. Temperature was maintained at 303K with the velocity‐rescaling thermostat [91] and a coupling constant of 1.0 ps^−1^. Energy minimization was performed using the steepest descent method, followed by NVT and NPT equilibration steps. Each system was simulated for a single 10 µs production run. Clustering analyses were performed using gmx clustsize command using a 1.0 nm cut‐off and 2D density maps were generated using gmx densmap using a grid size of 0.02 nm.

#### 5.9.5 RAB8b and VAMP‐3 in a POPC Bilayer

CG models of RAB8b and VAMP‐3 were obtained using the Martinize2 program [99], including the ‐scfix flag [100]. The OLIVES Go̅‐like model [101] was applied to stabilize the protein structures. In the control simulation, 25 VAMP‐3 molecules were placed in a 30 × 30 nm POPC bilayer (1400 lipids/leaflet), with their N‐terminal regions oriented upward (Supporting Information S1: Figure 7C, right), using the INSANE code [102]. For the RAB8b + VAMP‐3 system, four RAB8b molecules were positioned at the top edge of the simulation box, away from the VAMP‐3 molecules (Supporting Information S1: Figure 7C, left and center). A flat‐bottomed position restraint was applied to prevent RAB8b from crossing the simulation boundaries. Both systems were solvated and neutralized with Na+ and Cl− ions.

### 5.10 Coexpression Analysis

For coexpression analysis, the processed GSE174072 data set (https://covid19.cog.sanger.ac.uk/submissions/release1/blish_awilk_covid_seurat.rds) was downloaded and subsetted to COVID‐19 monocytes CD16. Pseudobulk expression profiles were reconstructed by aggregating raw counts across cell type‐samples using the “aggregateExpression” function in Seurat v4.3.0.1 [70]. Aggregated counts were normalized using the regularized log transformation from DESeq2 v1.40.2 [67]. Subsequently, CEMiTool v1.24.0 was employed to construct a signed coexpression network with a minimum of 30 genes per module and an automatically detected power beta [103].

For the second coexpression analysis, a Seurat object was set using the “fraction” method for gene selection, including only genes expressed in at least 5% of cells. Metacells were constructed by grouping cells based on donors, utilizing UMAP for dimensionality reduction, KNN with 25 nearest neighbors, and a maximum of 10 shared cells between 2 metacells. These metacells were processed using default parameters, and a coexpression signed network was constructed with a power beta of 9 using hdWGCNA v0.2.23 [104].

Lastly, the pySCENIC workflow was applied. The subset Seurat object for monocytes CD16 was converted into a Loom object using SeuratDisk v0.0.9020. A gene regulatory network was inferred, coexpression modules generated, and regulon cis targets predicted using pySCENIC v0.12.1 [105]. Auxiliary data sets included the cis target database (hg38_refseq_r80_v10_db_Gene_based ‐ Homo sapiens ‐ hg38 ‐ refseq_r80 ‐ SCENIC+ databases – Gene‐based (aertslab.org)), motif‐to‐transcription factor annotations (motifs‐v9‐nr.hgnc‐m0.001‐o0.0.tbl ‐ Motif2TF annotations (aertslab.org)), and the list of transcription factors (allTFs_hg38.txt ‐ (https://resources.aertslab.org/cistarget/tf_lists/allTFs_hg38.txt)), all retrieved from the Aerts Lab resources.

### 5.11 Lentiviral shRNA Cloning and Viral Production

Target sequences to shGFP (5‐CAAGCTGACCCTGAAGTTCAT‐3), shRAB8B_1 (5′‐GTCGTGAAGTTCTAGACAAAT‐3′, TRCN0000380248) shRAB8B_2 (5′‐CCTGGGTAACAAATGTGATAT‐3′, TRCN0000047879) and shOSBPL8 (5′‐CAGAGTTCCATCGAATCTATA‐3′, TRCN0000146765) were cloned into the AgeI and EcoRI restriction sites of pLKO.1‐puro lentiviral vector (Addgene #8453). HEK‐293 cell line was used for packing viral particles using psPAX2 (Addgene #12260) and pMD2.G (Addgene #12259) plasmids.

### 5.12 SARS‐CoV‐2 Infection

Immortalized cell line of human colorectal adenocarcinoma CaCo‐2 was maintained in DMEM low glucose supplemented with 20% fetal bovine serum and incubated at 37°C under 5% CO_2_ in a humidified atmosphere. CaCo‐2 cell line was transduced with lentiviral particles (MOI 0.1) from the pLKO.puro shGFP, shRab8b_1, shRab8b_2, or shOsbpl8. After a week of 1 μg/mL puromycin selection, experiments were performed in quadruplicate for each condition of CaCo‐2 transduction (shGFP, shRab8b_1, shRab8b_2, and sh), both in the presence and absence of SARS‐CoV‐2 infection. For the infection condition, cells were infected with the original SARS‑CoV‑2 lineage isolate (HIAE‑02 SARS‑CoV‑2/SP02/human/2020/BRA; GenBank accession MT126808.1) at MOI 0.1, as previously described [106]. After 24 h of infection, cells were collected with 200 µL of Trizol to total RNA extraction. cDNA reaction was performed using GoScript Reverse Transcriptase cDNA (Thermo Scientific – #4311235) synthesis kit according to the manufacturer's instructions. qPCR reactions were performed to viral load and knockdown confirmation. For viral load a standard curve was generated using serial dilutions of SARS‐CoV‐2 and SARS‐CoV‐2 N1 region‐specific primers were used (Forward 5′‐CAATGCTGCAATCGTGCTAC‐3′ and Reverse 5′‐GTTGCGACTACGTGATGAGG‐3′). For knockdown confirmation RAB8B (Forward 5′‐TTCCGATGTCGAAAGAATGATCC‐3′ and Reverse 5′‐TGCGCTTGTCTCCAAGAATTTA‐3′) and OSBPL8 (Forward 5′‐TGGCTGATTGGTTAAAGATTCGT‐3′ and Reverse 5′‐GCACCCCAGGTTTCAACACA‐3′) specific primers were used. All qRT‐PCR reactions were performed using QuantiNova SYBR Green PCR Kit (Qiagen − #208056). The controls used in the experiments included shGFP as the control for knockdown assays using shRNA, and noninfected cells as the control for SARS‐CoV‐2 infection. Statistical analysis was conducted using an unpaired *t*‐test, and differences were considered statistically significant at *p* < 0.05.

## Author Contributions

Data curation: all authors. Formal analysis: Amanda Pereira Vasconcelos, Adriana Simizo, Bianca G. Castelucci, Débora Guerra Peixe, Ícaro Castro, Jonathan Peña Avila, João Victor Virgillio‐da‐Silva, Larissa Menezes, Marcelo Berçot Rodrigues, Mauro César Cafundó Morais, Pedro M. Moraes‐Vieira, Peter Park, Thiago Dominguez Crespo Hirata, Thiago Lubiana, Thomaz Lüscher Dias, Viviane Schuch, Youvika Singh. Funding acquisition: Larissa Menezes, Pedro M. Moraes‐Vieira, Helder I. Nakaya. Investigation: Amanda Pereira Vasconcelos, Adriana Simizo, Bianca G. Castelucci, Débora Guerra Peixe, Jonathan Peña Avila, João Victor Virgillio‐da‐Silva, Larissa Menezes, Marcelo Berçot Rodrigues, Pedro M. Moraes‐Vieira, Peter Park, Thiago Dominguez Crespo Hirata, Thomaz Lüscher Dias, Youvika Singh, Helder I. Nakaya. Methodology: Amanda Pereira Vasconcelos, Adriana Simizo, Bianca G. Castelucci, Débora Guerra Peixe, Jonathan Peña Avila, João Victor Virgillio‐da‐Silva, Larissa Menezes, Marcelo Berçot Rodrigues, Pedro M. Moraes‐Vieira, Peter Park, Thiago Dominguez Crespo Hirata, Thomaz Lüscher Dias, Youvika Singh, Helder I. Nakaya. Project administration: Jonathan Peña Avila, Thomaz Lüscher Dias, Thiago Dominguez Crespo Hirata, Helder I. Nakaya. Database contributor: Rodrigo Aquino, Luiz Durão, Jeevan Giddaluru. Resources: Helder I. Nakaya. Writing – original draft: Jonathan Peña Avila, Peter Park. Writing – review and editing: Juan Carlo Santos e Silva, Jonathan Peña Avila, Pedro M. Moraes‐Vieira, Thiago Dominguez Crespo Hirata, Otavio Cabral‐Marques, Helder I. Nakaya. Supervision: Helder I. Nakaya.

## Conflicts of Interest

The authors declare no conflicts of interest.

## Supporting information

Supporting File 1

Supporting File 2

Supporting File 3

Supporting File 4

Supporting File 5

Supporting File 6

Supporting File 7

Supporting File 8

Supporting File 9

Supporting File 10

Supporting File 11

Supporting File 12

## Data Availability

A GitHub repository containing the scripts and workflows used in the analysis is available at: https://github.com/jonthpa/COVID-19_Transcriptome_Atlas. Molecular Dynamics simulations analysis is available at https://zenodo.org/records/16587626.
