# Supplementary material for: Multiorgan Molecular Landscape of Severe COVID‐19 Revealed by Consensus Gene Signatures and RAB8B Targeting
Source: J Med Virol. 2026 Apr 21;98(4):e70932. doi: 10.1002/jmv.70932 (PMC13100344; doi:10.1002/jmv.70932)
Supplement: Supplementary file 1 — Supporting File 1 [file JMV-98-e70932-s001.docx]

**
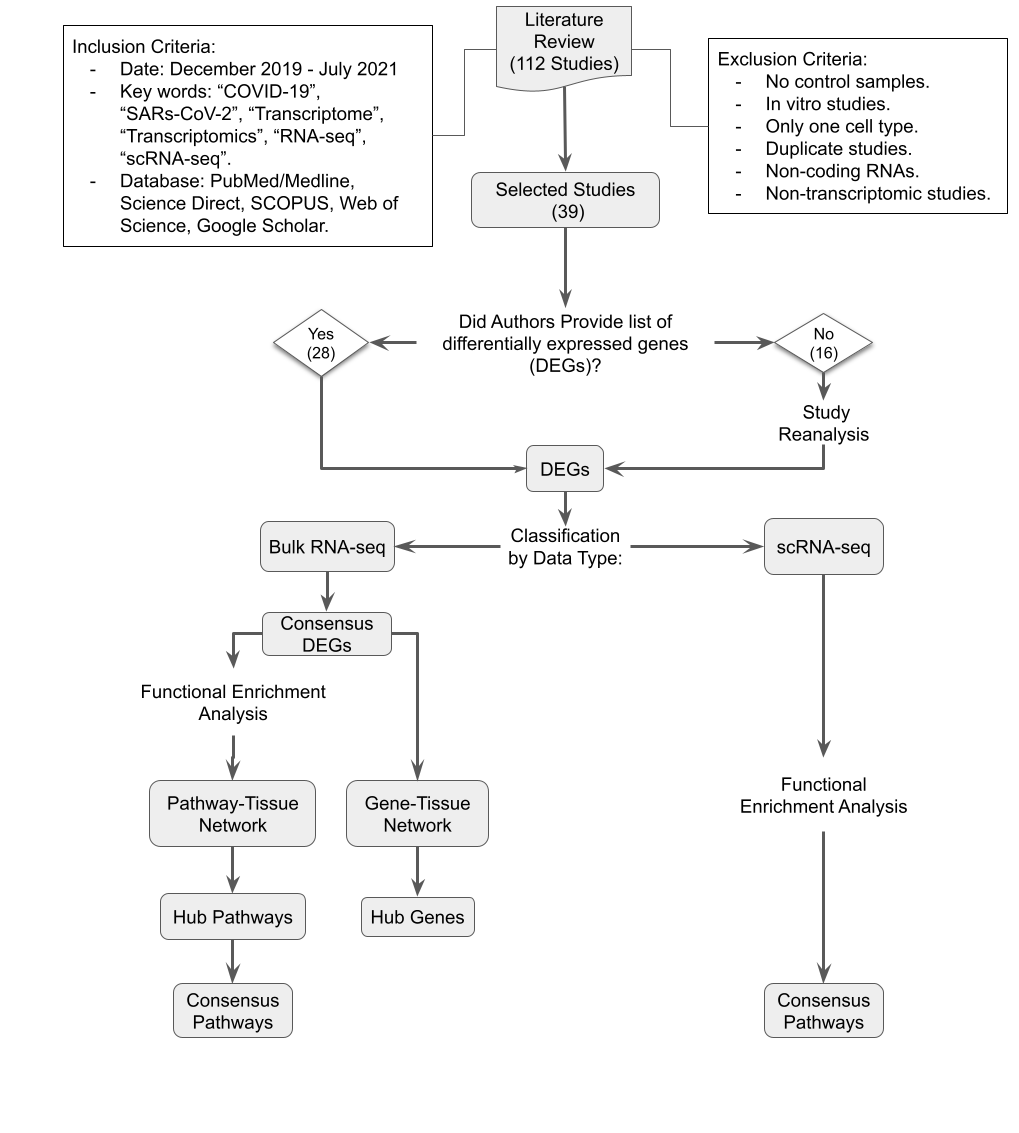
**

**Figure S1. Flowchart of Study Methods.** Flowchart of the project methods outlining the main steps involved in identifying consensus gene signatures and molecular mechanisms associated with COVID-19 patients.

**
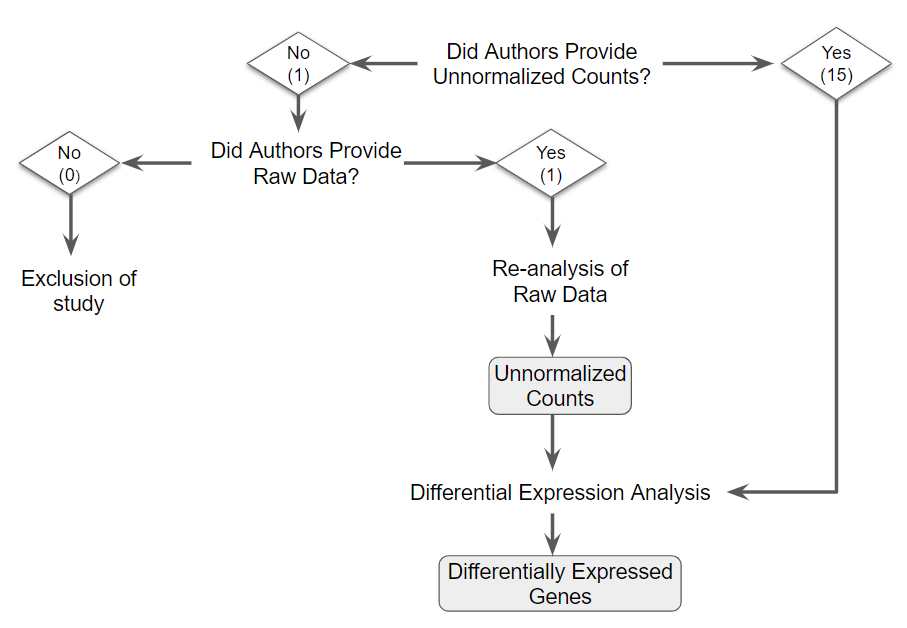
 Figure S2: Flowchart of Study Reanalysis Methodology.** This flowchart outlines the critical steps involved in the reanalysis of the study, specifically focusing on obtaining differentially expressed genes (DEGs) when the original authors did not provide lists of DEGs associated with COVID-19 patients.

*
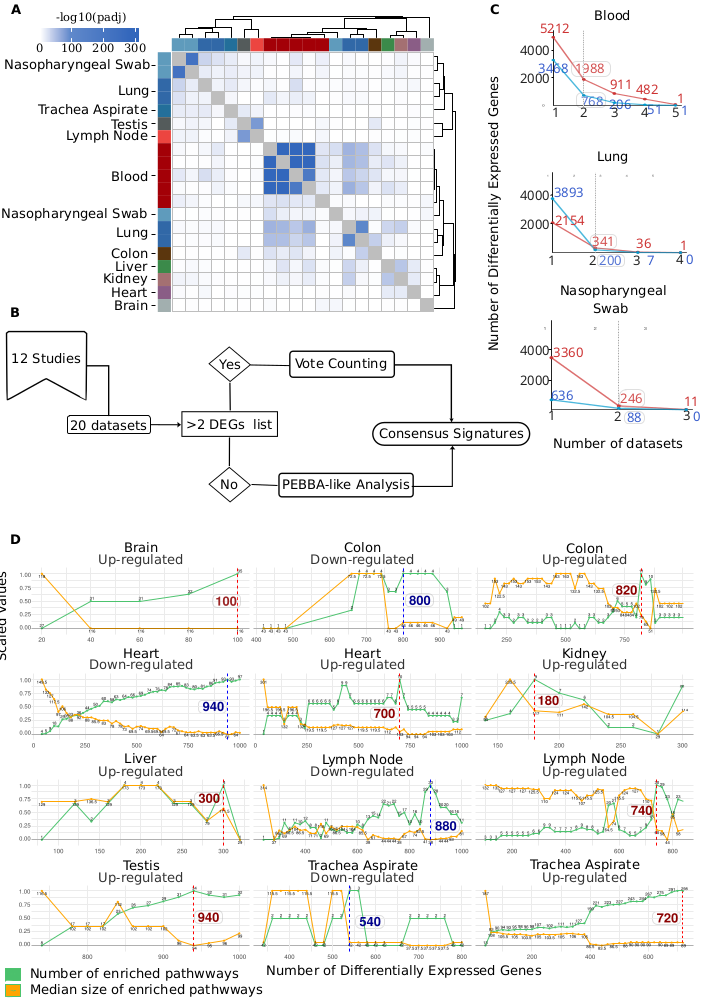
*

## **Figure S3: Consensus Gene Signatures Identification Methods. a.** Heatmap representation of Fisher test enrichment scores (-log10padj) for lists of differentially expressed genes (DEGs) in bulk-RNAseq data that contains all genes from original studies. Rows and columns depict the lists, highlighting the importance of a consensus analysis for robust gene signature identification across datasets. **b.** Schematic representation of the consensus analysis approach, utilizing vote counting methods when multiple lists of DEGs were available. A systematic iterative pathway enrichment analysis approach is employed in cases where only one list was collected. **c.** Line plot illustrating the accumulated distribution of DEGs across varying numbers of datasets. The x-axis represents the number of datasets, while the y-axis displays the total number of common DEGs. Blue and red lines represent downregulated and upregulated genes, respectively. **d.** Line plot illustrating the systematic iterative pathway enrichment analysis approach. The x-axis shows the number of DEGs used for enrichment analysis, while the y-axis displays scaled values for the number of enriched pathways (padj < 0.05) and the median size of enriched pathways. Green lines represent the number of enriched pathways, and yellow lines indicate the median size of enriched pathways. The dashed line represents the number of genes considered consensus signatures, with blue (downregulated) and red (upregulated) lines indicating the number of consensus DEGs. Numbers above the lines denote absolute values for each enrichment result.


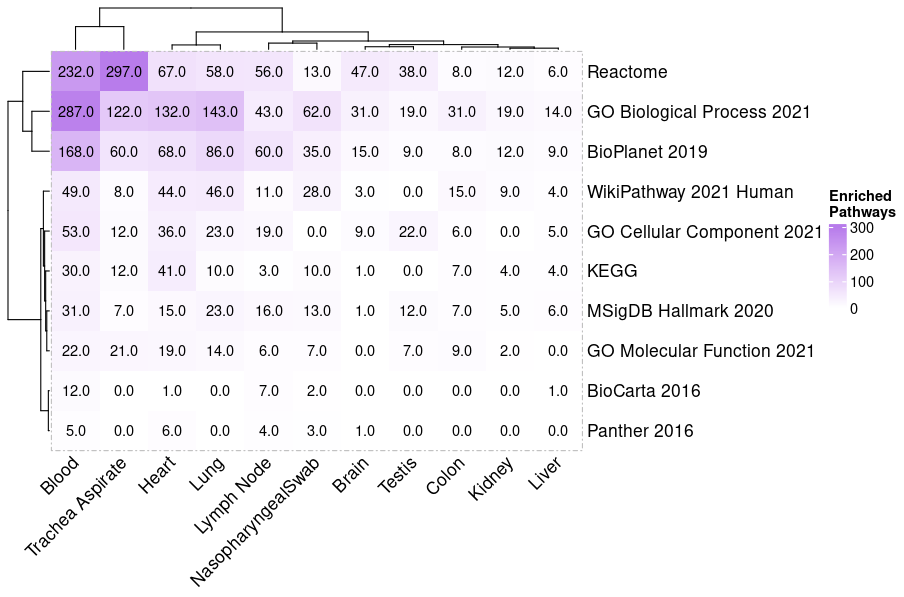
 **Figure S4: Enriched Signaling Pathways by Geneset in Patients with Severe COVID-19.** The number of significantly enriched pathways (P-adj < 0.05) in each tissue is illustrated using Fisher's Exact Test as the statistical method. The analysis incorporates various annotation databases, including "BioCarta 2016," "BioPlanet 2019," "GO Biological Process 2021," "GO Cellular Component 2021," "GO Molecular Function 2021," "MSigDB Hallmark 2020," "Panther 2016," and "WikiPathway_2021_Human." Color Intensity: The intensity of the color directly corresponds to the number of enriched pathways, allowing for a visual assessment of the level of enrichment.

## *
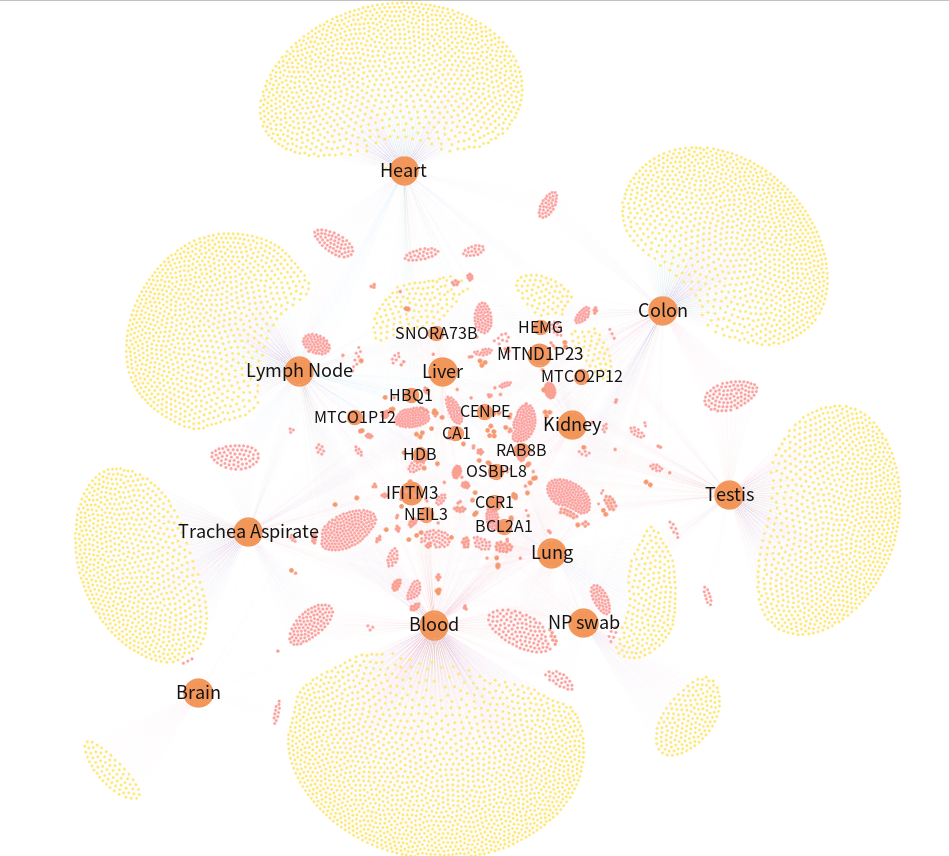
* **Figure S5. Network Analysis of consensus differentially expressed genes in severe COVID-19 in BulkRNAseq data.** Exploration of hub consensus differentially expressed genes (DEGs) through network analysis, where nodes represent consensus DEGs connected with tissues in bulk-RNAseq data. Nodes with a degree greater than or equal to 5 are highlighted, shedding light on central genes in severe COVID-19 across diverse tissues.

## **
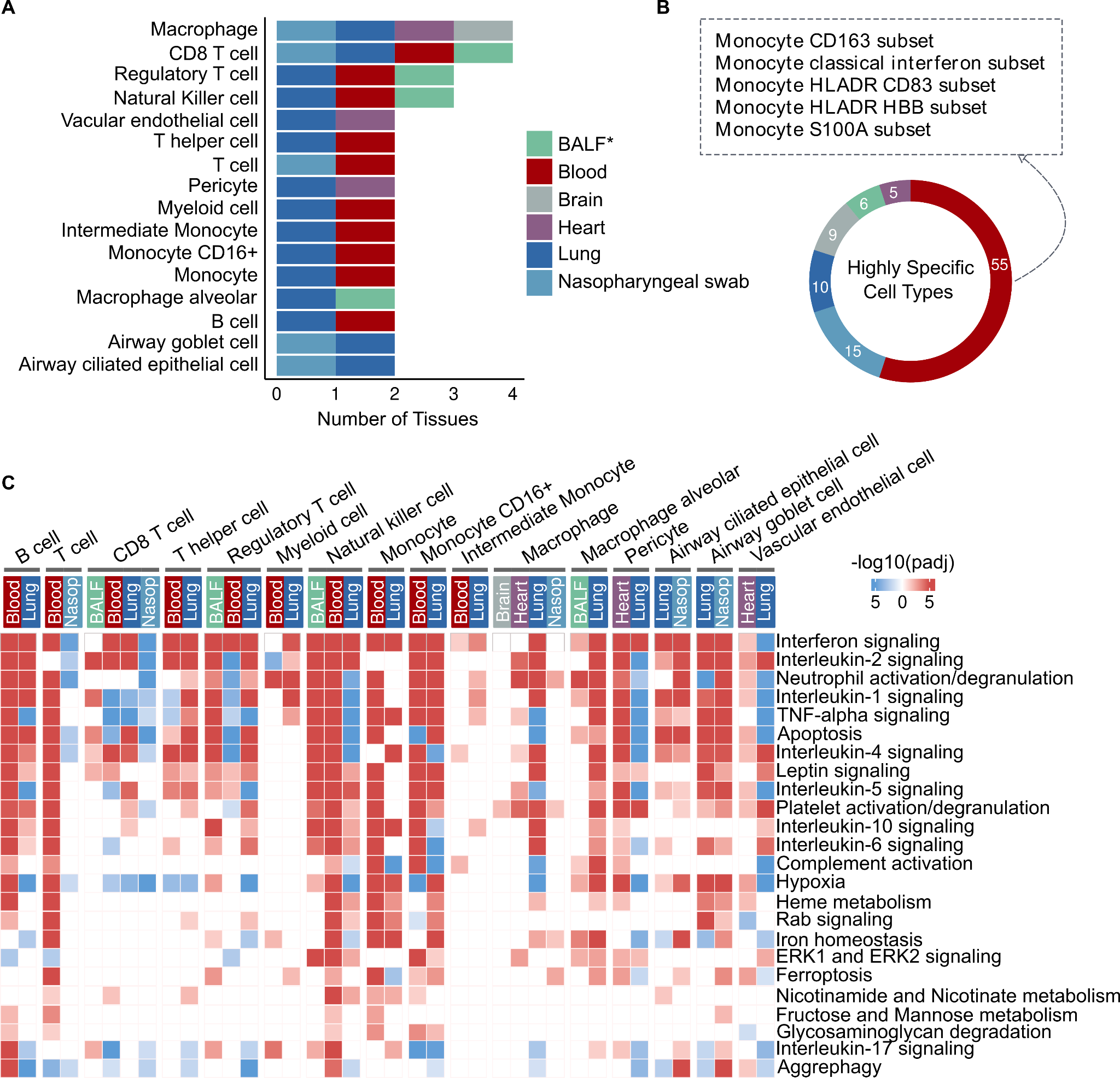
 Figure S6: Cell Types and Enriched Pathways in scRNAseq. a.** Barplot illustrating cell types identified in two or more tissues. The x-axis represents the number of tissues where each cell type was identified, while the y-axis denotes the cell types. **b.** Number of Highly Specific Cell Types in Each Tissue. The donut plot presents the number of particular cell types identified in each tissue. The box text highlights particular monocyte subsets identified in peripheral blood. **c.** Heatmap of enriched pathways in scRNAseq data, with a specific focus on their presence in bulkRNAseq data, as identified in previous analyses. Enrichment scores are color-coded, with blue indicating downregulated pathways and red indicating upregulated pathways. Top annotations specify the associated tissue and cell types, providing a comprehensive understanding of dysregulated pathways across different cell populations.


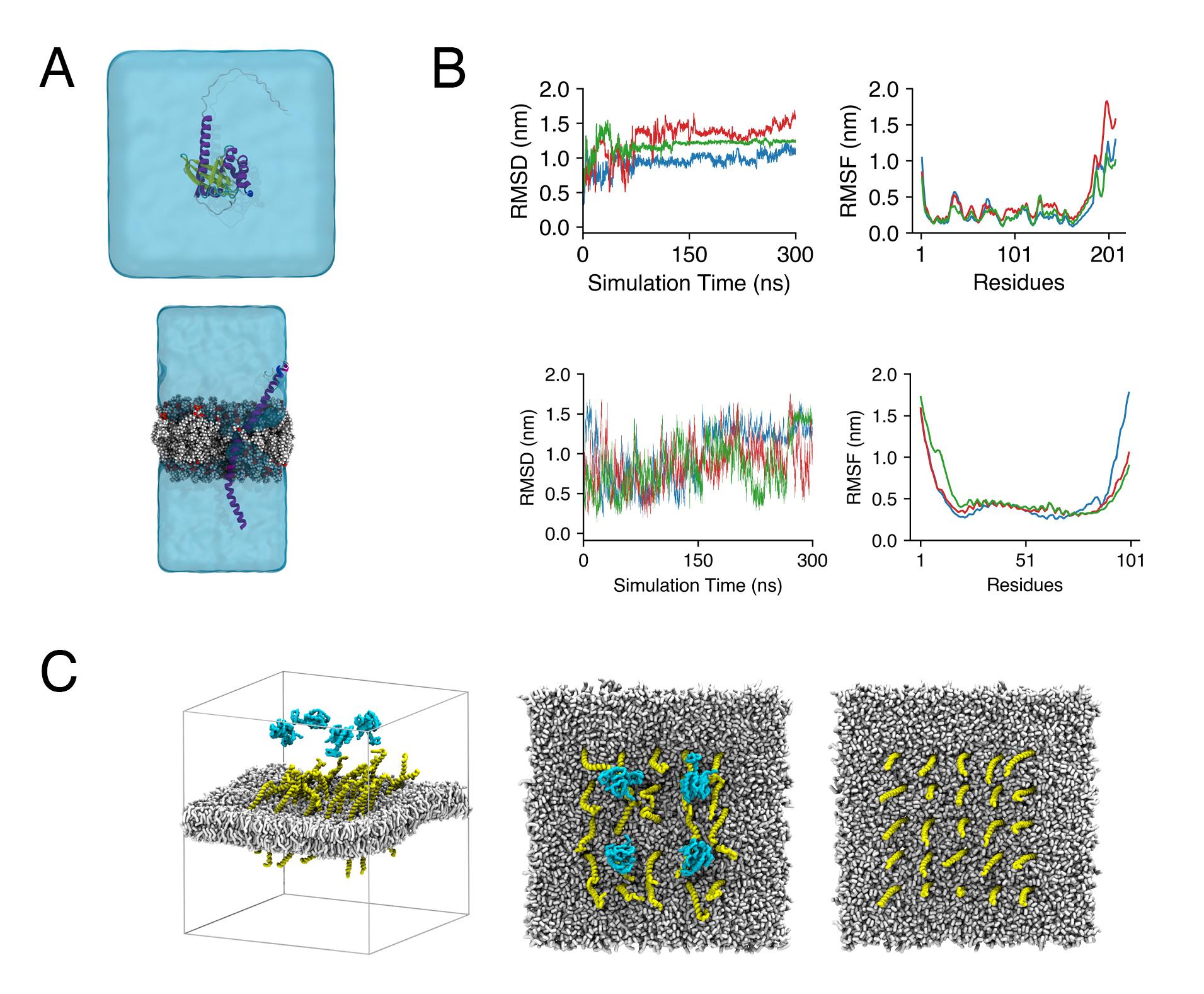
 **Figure S7: Atomistic and Coarse-Grained Simulation Setup:** **a.** Snapshots of the initial atomistic systems, RAB8b in water (top) and VAMP-3 in a POPC membrane (bottom). **b.** RMSD and RMSF of the three replica AA simulations (RAB8b on top, VAMP-3 on the bottom). **c.** Snapshots of the CG simulations: RAB8b + VAMP3 in POPC (left and center, side, and top view, respectively), and VAMP3 in POPC (right). RAB8b are depicted in cyan, VAMP-3 in yellow, and POPC lipids in silver.
