# Supplementary material for: Multiorgan Molecular Landscape of Severe COVID‐19 Revealed by Consensus Gene Signatures and RAB8B Targeting
Source: J Med Virol. 2026 Apr 21;98(4):e70932. doi: 10.1002/jmv.70932 (PMC13100344; doi:10.1002/jmv.70932)
Supplement: Supplementary file 12 — Supporting File 12 [file JMV-98-e70932-s011.docx]

**Multi-Organ Molecular Landscape of Severe COVID-19 Revealed by Consensus Gene Signatures and RAB8B Targeting**

###

Jonathan Peña Avila^1,2^*, Peter Park^1^*, Youvika Singh^1,10^, Paulo P. Amaral^3^, Ícaro Castro^4^, Felipe Ten-Caten^5^, Viviane Schuch^1^, André N.A. Gonçalves^1^, Jeevan Giddaluru^1^, Mauro César Cafundó Morais^1,2^, Rodrigo L.T Ogava^1^, Thiago Lubiana^1^, Gabriel Amoroso de Castro^6^, Rodrigo Aquino^1^, Luiz Durão^1^, Júlia Raspante Martins^7^, Leandro Jimenez^1^, André G. Costa-Martins^1,11^, Patrícia Gonzalez-Dias^1^, Thiago Dominguez Crespo Hirata^1^, Thomaz Lüscher Dias^1^, Débora Guerra Peixe^4^, Adriana Simizo^15^, Juan Carlo Santos e Silva^1,2^, Amanda Pereira Vasconcelos^1^, Marcelo Berçot Rodrigues^8,9^, Bianca G. Castelucci^8^, João Victor Virgillio-da-Silva, Larissa Menezes^8^, Pedro M. Moraes-Vieira^8^, Otavio Cabral-Marques^1,4,12-14^, Helder I. Nakaya^1,2,15^

^1^ Department of Clinical and Toxicological Analyses, University of São Paulo, São Paulo, SP, Brazil

^2^ Institut Pasteur de São Paulo, University of São Paulo, São Paulo, SP, Brazil

^3^ Instituto de Ensino e Pesquisa, Insper, São Paulo, Brazil

^4^ Interunit Postgraduate Program on Bioinformatics, Institute of Mathematics and Statistics, University of São Paulo, São Paulo, SP, Brazil

^5^ Department of Infectious and Parasitic Diseases, University of São Paulo Medical School, University of São Paulo, São Paulo, Brazil

^6^ Department of Pharmaceutical Sciences, State University of Campinas, Campinas, Brazil

^7^ Department of Pharmaceutical Sciences, University of São Paulo, Ribeirão Preto, SP, Brazil

^8^ Department of Genetics, Microbiology and Immunology, Institute of Biology, State University of Campinas, Campinas, Brazil

^9^ Department of Immunology, Institute of Biomedical Sciences, University of São Paulo, São Paulo, SP, Brazil

^10^ Department of Parasitology, Leiden University Center for Infectious Diseases (LU-CID), Leiden University Medical Center, Leiden, The Netherlands

^11^ Micro Manufacturing Laboratory, Institute for Technological Research - IPT, São Paulo, SP, Brazil

^12^ Department of Medicine, Division of Molecular Medicine, Laboratory of Medical Investigation, University of São Paulo School of Medicine, São Paulo, SP, Brazil

^13^ Instituto D'Or de Ensino e Pesquisa, São Paulo, Brazil

^14^ Network of Immunity in Infection, Malignancy, Autoimmunity (NIIMA), Universal Scientific Education and Research Network (USERN), São Paulo, SP, Brazil

^15^ Hospital Israelita Albert Einstein, São Paulo, SP, Brazil

*These authors contributed equally to this work.

Corresponding author: Hospital Israelita Albert Einstein, São Paulo 05652-900, Brazil

Email address: [hnakaya@usp.br](mailto:hnakaya@usp.br) (Helder I. Nakaya)

# **SUPPLEMENTARY METHODS**

## Study selection and transcriptome data collection

A comprehensive literature review was conducted, initially identifying 112 studies. We included studies that provided transcriptomic data (bulk RNA-seq or scRNA-seq) from human samples related to COVID-19; included samples from relevant tissues such as lung, blood, brain, heart, and kidney; reported data from patients with clearly defined clinical severity, particularly those with severe or critical COVID-19; had available lists of differentially expressed genes (DEGs) or raw data suitable for reanalysis; were published between December 2019 and July 2021; and provided sufficient metadata to support interpretation, including tissue type, disease status, and experimental conditions. Studies were excluded if they lacked patient-level metadata for disease severity categorization, had insufficient sequencing depth or quality, or used only in vitro or non-human models. Additional exclusion criteria included absence of control samples, analysis limited to a single cell type, lack of publicly deposited raw data, duplicate publications, focus on non-coding RNAs, or if the study was not based on transcriptomic data. After applying these criteria, 39 studies were selected for further analysis (Figure S1). Lists of DEGs from COVID-19 patients and control cases were compiled, adhering to the significance cutoff values provided by the study authors. Alongside DEGs, relevant metadata were extracted, including details on comparisons, group descriptions, sample sizes, tissue and cell types, and statistical tests. Statistical cutoff values for log2FoldChange, p-values, and adjusted p-values were recorded. The sequencing type - Bulk-RNA (bulk RNA-seq) or single-cell RNA sequencing (scRNA-seq) - and study attributes, including titles, access links, codes, and data repository information, were documented, ensuring transparency in our analytical processes.

**
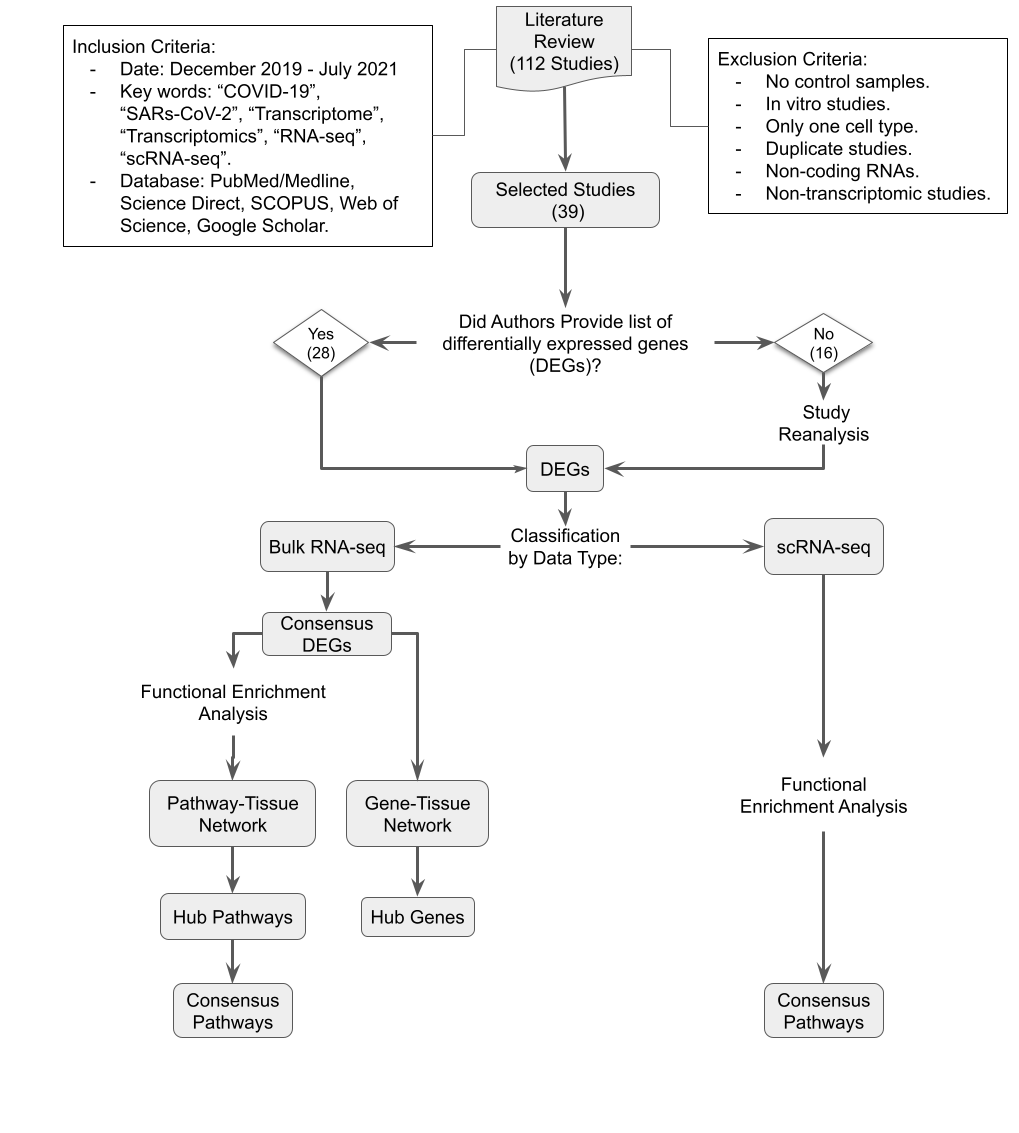
**

**Figure S1. Flowchart of Study Methods.** Flowchart of the project methods outlining the main steps involved in identifying consensus gene signatures and molecular mechanisms associated with COVID-19 patients.

## Reanalysis of bulk RNA-seq studies

- 1. Reanalysis of Raw Data

In cases where lists of DEGs and raw count matrices were unavailable, raw RNA sequencing data were retrieved from the Gene Expression Omnibus (GEO). FASTQ files were downloaded using SRA Toolkit v2.10.9 [[1]](https://www.zotero.org/google-docs/?eYs9aB). For Quality control analysis, we filtered out sequences with Phred scores below 30, sequences shorter than 36 bases, and the adapter sequences removed using FASTP v0.23.2. [[2]](https://www.zotero.org/google-docs/?HEwd8p). Reads passing quality control were aligned and mapped to the *Homo sapiens* reference genome (Homo_sapiens.GRCh38.dna.primary_assembly.fa) and gene model (Homo_sapiens.GRCh38.104.gtf), using STAR v2.7.9a default parameters [[3]](https://www.zotero.org/google-docs/?K2NVHQ). The integrity and quality of the reads before and after preprocessing were assessed using FastQC v0.11.9, and a summary of quality metrics across samples was compiled using MultiQC v1.12 (Figure S2) [[4,5]](https://www.zotero.org/google-docs/?na11As).

- 1. Differential Expression Analysis

For studies lacking DEG tables, differential expression analysis was conducted using raw count tables from re-sequencing or original studies (Figure S2). This process involved removing genes with zero counts, identifying outliers using Cook's distances and MDP scores, and employing DESeq2 v1.40.2. [[6,7]](https://www.zotero.org/google-docs/?IzcFOU). We applied a standard statistical cutoff of an adjusted p-value (padj​) < 0.01 and an absolute log2-fold change (∣log2​FC∣) > 1. An exception was made for study number 62, which focused on the brain. Here, we applied a padj​ < 0.05 without a log2​FC threshold. Gene identifiers were converted from ENSEMBL IDs to Gene Symbols using BioMart v2.58.0, and ggplot2 v3.4.4 was used for visualization [[8,9]](https://www.zotero.org/google-docs/?mlp72w).

## **
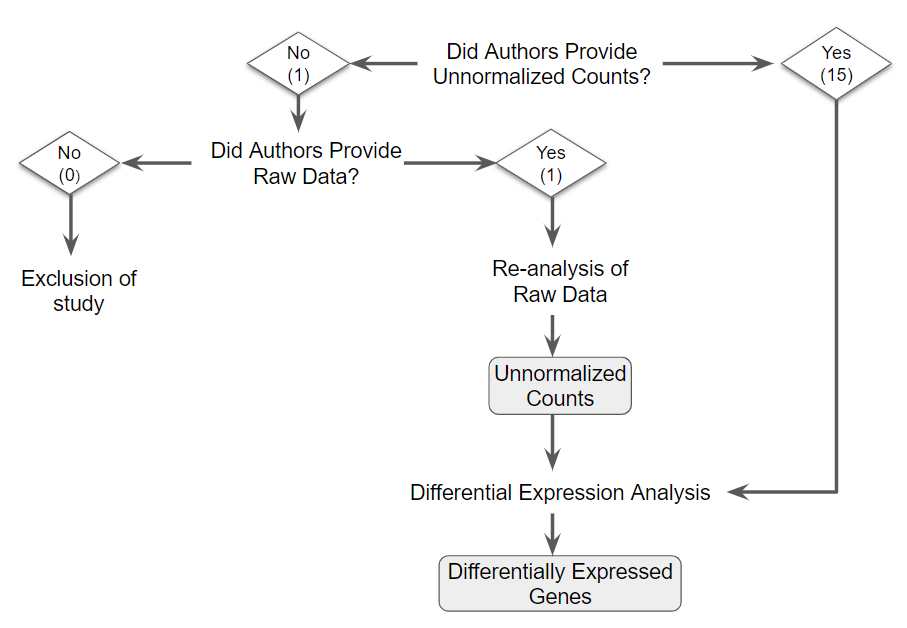
Figure S2: Flowchart of Study Reanalysis Methodology.** This flowchart outlines the critical steps involved in the reanalysis of the study, specifically focusing on obtaining differentially expressed genes (DEGs) when the original authors did not provide lists of DEGs associated with COVID-19 patients.

## Reanalysis of scRNA-seq studies

In studies where the authors did not provide lists of DEGs, available matrices from the 10x Genomics platform were used, including matrix.mtx, genes.tsv (or features.tsv), and barcodes.tsv. The subsequent analyses followed the standard Seurat v4.3.0.1 workflow. First, quality control was performed by excluding cells with fewer than 200 detected genes or more than 10% mitochondrial gene content. The data were then log-normalized using the NormalizeData() function with default parameters. Highly variable features were identified using FindVariableFeatures(), and the top 2000 highly variable genes were used to scale the data with ScaleData() and to perform dimensionality reduction using principal component analysis (PCA). Clustering was conducted through neighborhood graph construction and clustering using the FindNeighbors() and FindClusters() functions, applying a resolution of 0.5 and using the first 20 principal components. For visualization, UMAP was computed using the RunUMAP() function, also with the first 20 dimensions. Cell type annotation was performed manually using the marker genes described in the corresponding original publications. Differential expression analysis was conducted using the FindMarkers() function, comparing COVID-19 samples to controls for each identified cell type, with the following parameters: Wilcoxon statistical test and a logFC threshold of 0.25 [[10,11]](https://www.zotero.org/google-docs/?Thwy4g). Since each dataset was processed separately and under the same experimental conditions, no batch correction was required.

## Identification of consensus gene signatures in bulk RNA-seq data.

The lists of DEGs were categorized into groups by sequencing method, tissue, and cell type. First, all lists of DEGs were filtered for statistical significance. When lists of DEGs were provided by the original authors, we adhered to the thresholds reported in their respective publications to preserve the context and integrity of the original analyses. For datasets that lacked the lists of DEGs and required reanalysis, we applied a standardized statistical cutoff: adjusted p-value (padj) < 0.01 and absolute log2FC (|log2FC|) > 1. One exception was dataset #62 (brain tissue), where we used padj < 0.05 without a log2FC cutoff. This exception was necessary due to the low number of DEGs detected with more stringent thresholds, which would have risked excluding biologically relevant signals in a tissue with inherently subtle expression changes.

For groups with multiple lists of DEGs derived from bulk RNA‑seq data, consensus DEGs were identified using a vote‑counting approach in which genes exhibiting an absolute sum of expression-direction votes of two or more were retained. These consensus DEGs were then analyzed further: median log2FC values were calculated to assess gene expression trends, and combined p‑values were determined using Fisher’s method to evaluate statistical significance across multiple studies. For instance, if a gene was a DEG in a given tissue in three studies and in one study downregulated, the vote sum would be +3 (4 − 1), indicating a consensus for upregulation. In this case, only the median of log2FC and the combined adjusted p-values from the three upregulated results were computed.

In groups with a single list of DEGs, a systematic method was applied. DEGs were classified as up-regulated or down-regulated, then ranked based on a score calculated as score = abs(log2FC)*-log2(padj), prioritizing genes with significant fold changes and statistical significance. The top 20 DEGs by score underwent enrichment analysis using Reactome and KEGG databases, employing the 'fora' function from FGSEA v1.26.0 and all human protein-coding genes from ENSEMBL. Pathways with 15 to 500 genes were targeted for significant relevance [[12,13]](https://www.zotero.org/google-docs/?7WEbAo).

Biologically relevant pathways were identified by considering those with padj < 0.05 and at least five gene overlaps, ensuring statistical and practical significance. The number of DEGs analyzed increased iteratively by 20, up to 1000, to explore gene sets and assess pathway enrichment stability. All enrichment results were compiled to determine the total number of enriched pathways and median size for each tissue and expression direction, determining consensus DEGs based on the highest total number of enriched pathways and the smallest median size (Figure S3D and [Table S2](https://docs.google.com/spreadsheets/d/1FeFOfltR5Dy1nDsUamA7rCiw-MF61omr/edit?usp=sharing&ouid=104315441070245750811&rtpof=true&sd=true)).

##

## *
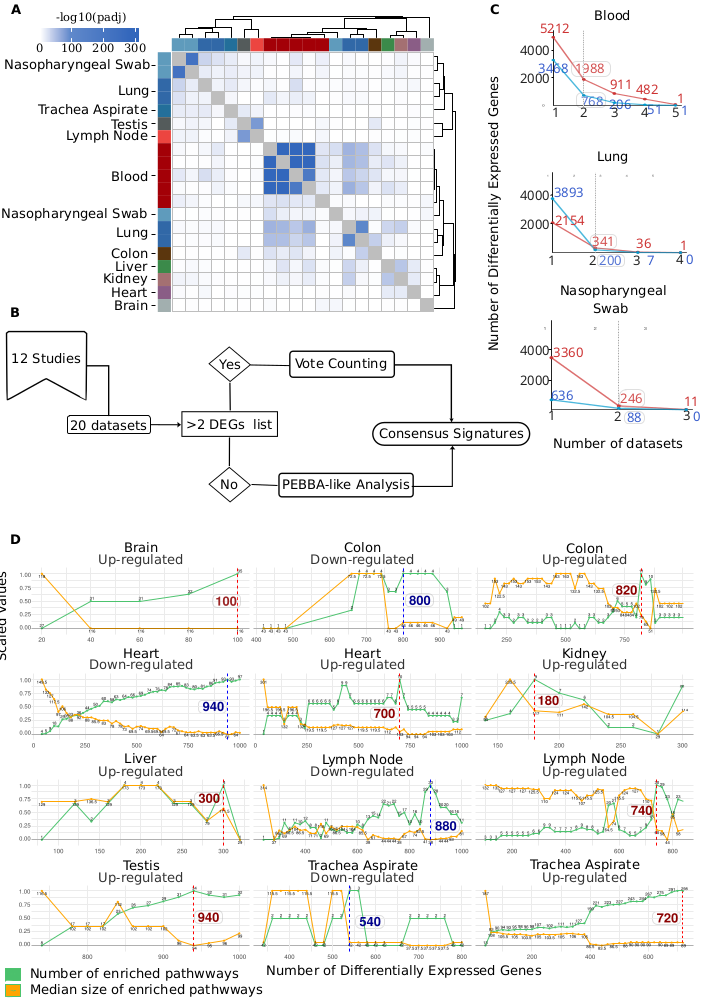
*

## **Figure S3: Consensus Gene Signatures Identification Methods. a.** Heatmap representation of Fisher test enrichment scores (-log10padj) for lists of differentially expressed genes (DEGs) in bulk-RNAseq data that contains all genes from original studies. Rows and columns depict the lists, highlighting the importance of a consensus analysis for robust gene signature identification across datasets. **b.** Schematic representation of the consensus analysis approach, utilizing vote counting methods when multiple lists of DEGs were available. A systematic iterative pathway enrichment analysis approach is employed in cases where only one list was collected. **c.** Line plot illustrating the accumulated distribution of DEGs across varying numbers of datasets. The x-axis represents the number of datasets, while the y-axis displays the total number of common DEGs. Blue and red lines represent downregulated and upregulated genes, respectively. **d.** Line plot illustrating the systematic iterative pathway enrichment analysis approach. The x-axis shows the number of DEGs used for enrichment analysis, while the y-axis displays scaled values for the number of enriched pathways (padj < 0.05) and the median size of enriched pathways. Green lines represent the number of enriched pathways, and yellow lines indicate the median size of enriched pathways. The dashed line represents the number of genes considered consensus signatures, with blue (downregulated) and red (upregulated) lines indicating the number of consensus DEGs. Numbers above the lines denote absolute values for each enrichment result.

## Testing robustness of consensus signatures.

Given the variability in sequencing platforms and other technical factors across datasets, we evaluated the representativeness of identified consensus DEGs compared to original signatures. Genes consistently differentially expressed across independent studies are more likely to reflect shared biological processes, whereas study-specific, spurious signals are less likely to overlap [[14]](https://www.zotero.org/google-docs/?TUHwoN). Then, to account for biological and technical heterogeneity, we classified lists of DEGs by tissue type, sequencing method, and clinical condition (severe COVID-19). We hypothesized that a robust consensus signature would show a higher degree of gene overlap within these stratified categories.

For this analysis, enrichment scores (-log10 p-value) for shared genes and enriched pathways between each list of DEGs were calculated using Fisher's exact test, implemented with the 'fora' function from the clusterProfiler v4.9.0.002. [[15]](https://www.zotero.org/google-docs/?qzwpRT). These scores measured the significance of gene overlap across different lists of DEGs. A symmetric matrix based on these scores was then constructed, followed by hierarchical cluster analysis using Pearson's correlation for distance measurement and the average agglomeration method. The results were visually displayed using ComplexHeatmap v2.16.0. [[16]](https://www.zotero.org/google-docs/?prToVa).

The concordance between original gene signatures and consensus DEGs was assessed by comparing the hierarchical grouping outcomes. This comparison involved lists of DEGs with all genes from original studies against those with only consensus DEGs and their enriched pathways. This approach provided insights into the consistency of clustering patterns and the extent to which consensus DEGs reflected the original signatures.

## Functional Enrichment Analysis

Overrepresentation analysis of consensus DEGs from bulk RNA-seq and individual gene signatures from scRNA-seq studies—separately for upregulated and downregulated genes—was performed using Fisher’s exact test. A wide range of annotation datasets was employed, including ‘Reactome 2022’, 'BioCarta 2016,' 'BioPlanet 2019,' 'GO Biological Process 2021,' 'GO Cellular Component 2021,' 'GO Molecular Function 2021,' 'MSigDB Hallmark 2020,' 'Panther 2016,' and 'WikiPathway 2021 Human' [[17–23]](https://www.zotero.org/google-docs/?gcq7WJ). All human protein-coding genes from the ENSEMBL database were the reference universe, ensuring comprehensive coverage. Enrichment analysis was focused on pathways with 15 to 500 genes for robustness and relevance. Pathways with an adjusted p-value below 0.05 were considered as statistically significant and included in the analysis. These analyses were conducted using FGSEA v1.26.0 and hypeR v2.0.1 ([Table S3](https://docs.google.com/spreadsheets/d/1Z1QNkW5nkYLwD2r2ac_8ENXYNCi2AR8q/edit?usp=sharing&ouid=104315441070245750811&rtpof=true&sd=true), and Figure S4 ) [[12,24,25]](https://www.zotero.org/google-docs/?QxT4P4).


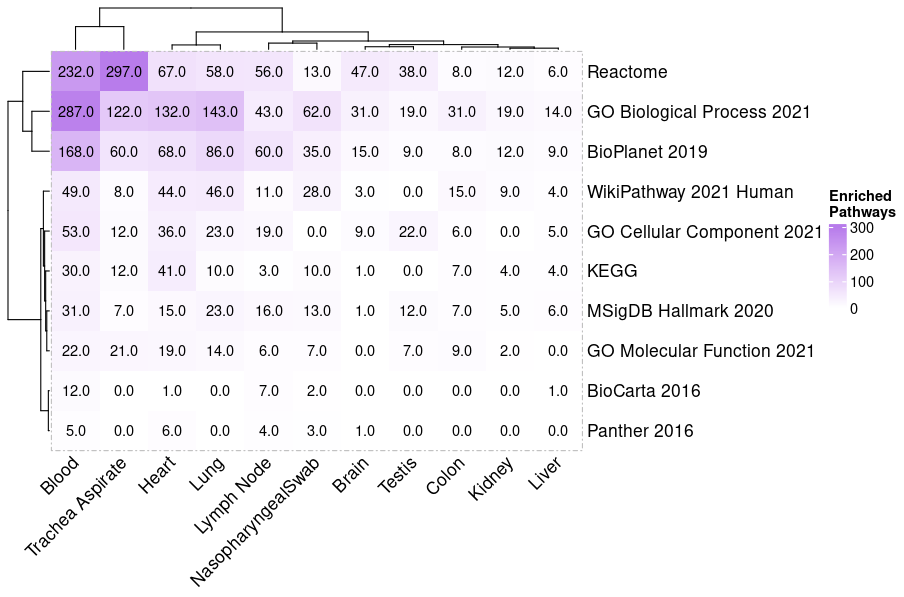


**Figure S4: Enriched Signaling Pathways by Geneset in Patients with Severe COVID-19.** The number of significantly enriched pathways (P-adj < 0.05) in each tissue is illustrated using Fisher's Exact Test as the statistical method. The analysis incorporates various annotation databases, including "BioCarta 2016," "BioPlanet 2019," "GO Biological Process 2021," "GO Cellular Component 2021," "GO Molecular Function 2021," "MSigDB Hallmark 2020," "Panther 2016," and "WikiPathway_2021_Human." Color Intensity: The intensity of the color directly corresponds to the number of enriched pathways, allowing for a visual assessment of the level of enrichment.

## Integrative analysis of consensus signatures and enriched pathways in bulk RNA-seq

Network analyses were performed on consensus DEGs and functional enrichment data from bulk RNA-seq studies, resulting in two network types to elucidate biological interactions: 1) Gene-Tissue Network: in this network, nodes represent consensus DEGs and tissues; an undirected, unweighted edge connects a consensus DEG to a tissue if the gene was identified as a consensus DEG in that tissue. 2) Pathway-Tissue Network: in this network, nodes represent enriched pathways and tissues; edges connect a tissue to a given pathway if it was found as significantly enriched (FDR ≤ 0.05) in that tissue. Edge weights correspond to –log₁₀(FDR), but all network metrics were computed on the unweighted graph. In both networks the layouts were generated using the Fruchterman Reingold (Area=800, Gravity=0.5, Velocity=1.0) algorithm, node size was mapped to degree and node color to modularity class (Figure S5).

Fundamental network properties such as density, diameter, degree, degree distribution, clustering coefficient, and centrality measures (betweenness, closeness, and eigenvector centrality) were calculated to characterize and interpret these networks. In the Gene-Tissue Network, a threshold was set for node degree identifying genes with a degree greater than four as the most representative across tissues. This implies that these genes show alterations in at least 45% of the analyzed tissues, marking them as crucial in the network. Gephi v0.10.1 software was utilized for robust network construction and analysis [[26]](https://www.zotero.org/google-docs/?fOnlzA). This comprehensive approach allowed us to identify not only the most interconnected genes across tissues but also the pathways most consistently dysregulated, providing insight into shared and tissue‑specific mechanisms of disease.

*
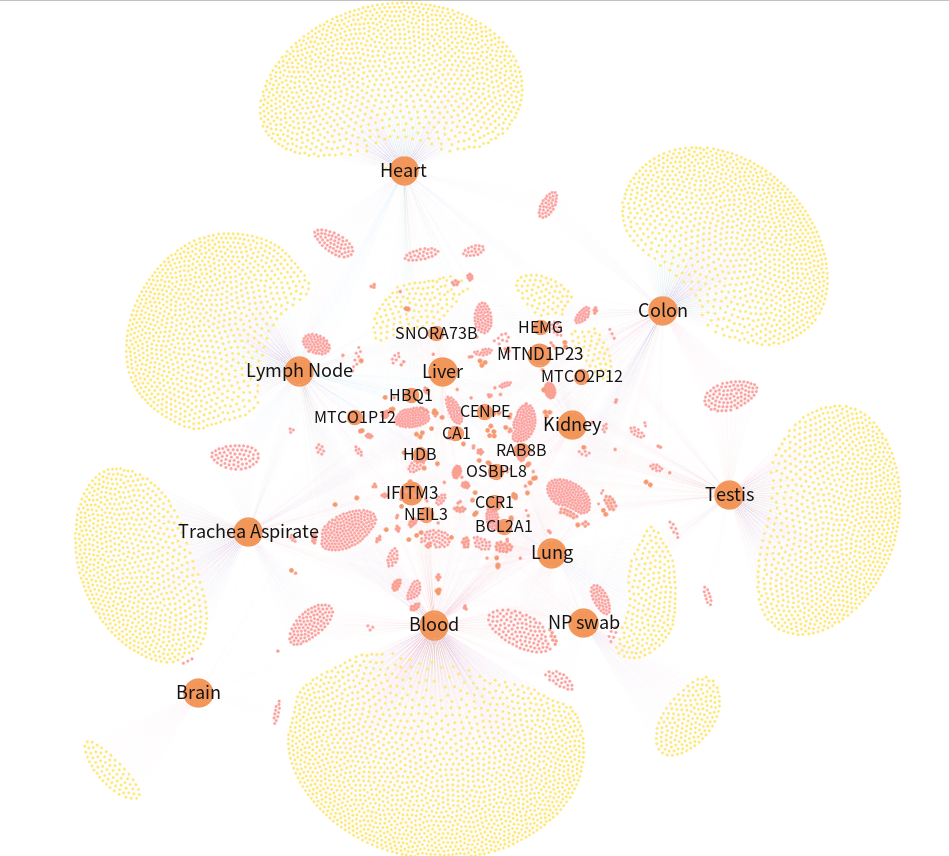
*

## **Figure S5. Network Analysis of consensus differentially expressed genes in severe COVID-19 in BulkRNAseq data.** Exploration of hub consensus differentially expressed genes (DEGs) through network analysis, where nodes represent consensus DEGs connected with tissues in bulk-RNAseq data. Nodes with a degree greater than or equal to 5 are highlighted, shedding light on central genes in severe COVID-19 across diverse tissues.

## Functional Enrichment Analysis of Selected Biological Processes using Consensus Gene Sets

A tailored approach was implemented to minimize redundancy in the functional enrichment results. A custom collection of gene sets, referred to as Consensus Gene Sets, was constructed by extracting genes from curated gene sets whose titles matched predefined keywords. We first compiled a list of keywords corresponding to biological processes relevant to our study. These processes were considered relevant based on prior literature evidence, their prominence in our network analysis (node degree > 5), or their exclusive association with a single tissue, suggesting tissue specificity.

The selected processes included immune-related pathways (e.g., complement activation, interferon signaling, cytokines such as interleukins and TNF-α), cellular stress responses (e.g., hypoxia, apoptosis, ferroptosis), metabolic and nutrient-related pathways (e.g., heme metabolism, vitamin and mineral homeostasis), and additional signaling cascades (e.g., ERK1/2, Hippo signaling, Rab GTPase activity).

We loaded pre-annotated gene sets containing protein-coding genes from curated resources, including BioCarta 2016, BioPlanet 2019, GO Biological Process 2021, GO Cellular Component 2021, GO Molecular Function 2021, MSigDB Hallmark 2020, Panther 2016, and WikiPathway_2021_Human. For each biological process, we performed a case-insensitive search for matching terms within gene set names using regular expressions. All gene sets with names matching any of the predefined process-related terms were selected. From these, we extracted and merged all associated genes, retaining only unique gene symbols per process.

This strategy allowed us to define a set of gene modules representing each biological process, while minimizing redundancy by aggregating overlapping terms and pathways. The resulting gene sets were used in downstream analyses including functional enrichment analysis applied to both bulk RNA-seq and scRNA-seq data using this customized annotation dataset, employing Fisher's exact test. The dataset was meticulously curated to include unique, protein-coding genes pertinent to the targeted pathways. Protein-coding genes from the ENSEMBL database were used as the reference universe for this analysis. Up-regulated and down-regulated genes were analyzed separately in the enrichment process. An adjusted p-value threshold of ≤ 0.05 was used to determine the statistical significance of the enriched pathways, ensuring a focused and relevant interpretation of the data.These analyses were conducted using FGSEA v1.26.0 and hypeR v2.0.1 [[12,24,25]](https://www.zotero.org/google-docs/?uyMc5b).

## Integrative Analysis of scRNA-seq data

To address heterogeneity in cell type annotation across studies, we implemented a hierarchical ontological framework for cell classification within each tissue, based on the Cell Ontology from Wikidata and manual curation of author-provided labels [[27]](https://www.zotero.org/google-docs/?KgE6eL).

For each scRNA-seq study, pathway enrichment analyses were performed separately for upregulated and downregulated genes, as described in Section 6, using overrepresentation analysis with an adjusted p-value cutoff of < 0.05 for significance and FGSEA v1.26.0. The enrichment direction (upregulated or downregulated) was defined according to the direction of the input gene list. In cases where the same pathway was significantly enriched (adjusted p-value < 0.05) for both upregulated and downregulated genes within a given cell type or subset, the direction was assigned based on the lowest adjusted p-value.

To determine the consensus direction of enrichment across cell types, a vote-counting strategy was applied. Each enrichment instance contributed a vote: +1 if the pathway was enriched in the upregulated gene set and −1 if in the downregulated gene set. A consensus direction was assigned to a given cell type or subset when the sum of votes was ≥ 2 or ≤ −2. For these consistent cases, adjusted p-values from studies with the same direction were combined using Fisher’s method. Only studies supporting the consensus direction were included in the combined p-value calculation. For instance, if a pathway was enriched in a given cell type (e.g., cell type A) in three studies using upregulated genes and in one study using downregulated genes, the vote sum would be +2 (3 − 1), indicating a consensus for upregulation. In this case, only the adjusted p-values from the three upregulated results were used in the Fisher’s method.

Importantly, results from cell subsets were not aggregated to infer consensus enrichment for broader (coarse) cell type categories. Finally, shared cell types or subsets across tissues were identified and used to compare enrichment scores of the same cell type across different tissues (Figure S6A–C).

## **
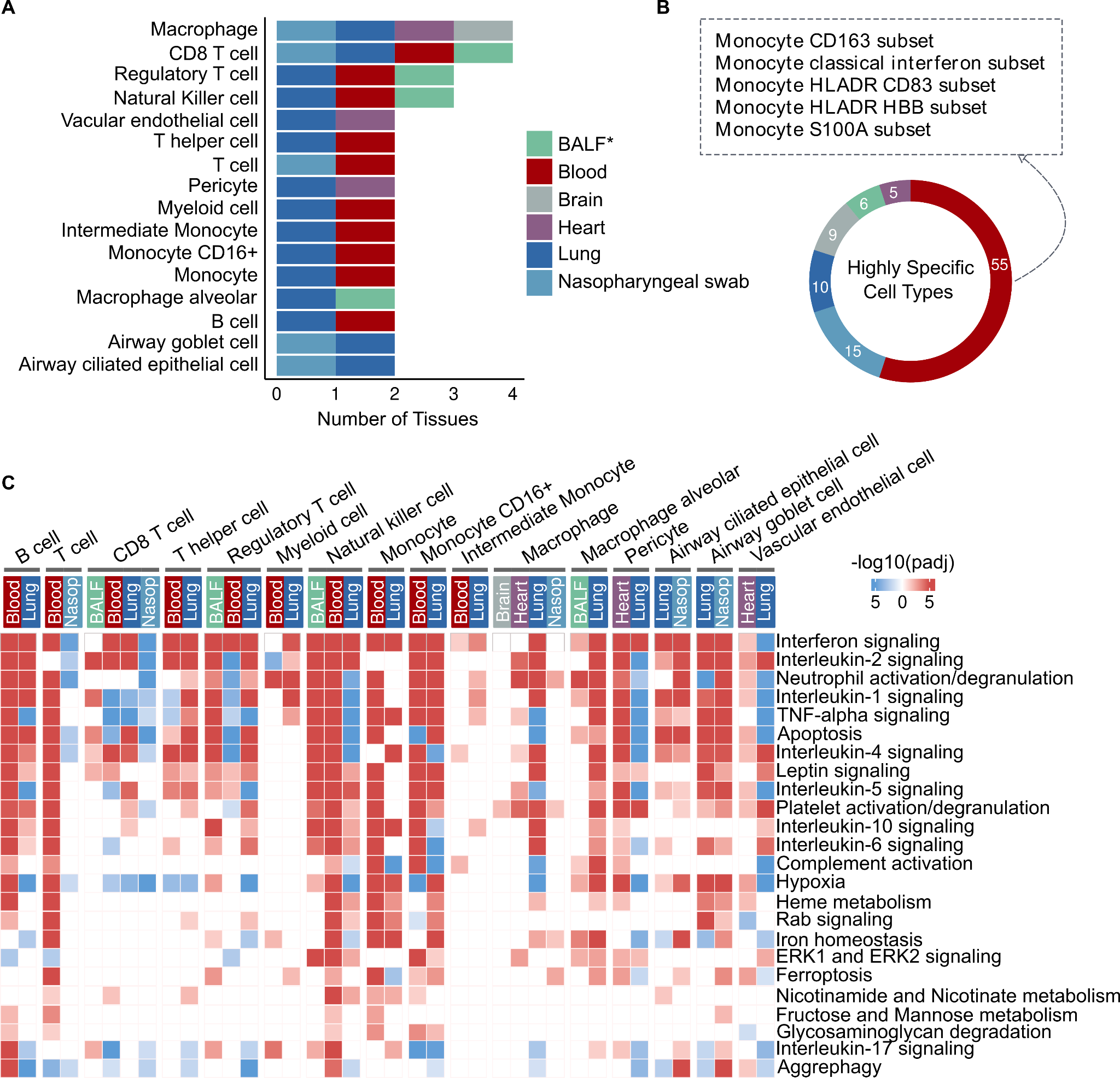
**

## **Figure S6: Cell Types and Enriched Pathways in scRNAseq. a.** Barplot illustrating cell types identified in two or more tissues. The x-axis represents the number of tissues where each cell type was identified, while the y-axis denotes the cell types. **b.** Number of Highly Specific Cell Types in Each Tissue. The donut plot presents the number of particular cell types identified in each tissue. The box text highlights particular monocyte subsets identified in peripheral blood. **c.** Heatmap of enriched pathways in scRNAseq data, with a specific focus on their presence in bulkRNAseq data, as identified in previous analyses. Enrichment scores are color-coded, with blue indicating downregulated pathways and red indicating upregulated pathways. Top annotations specify the associated tissue and cell types, providing a comprehensive understanding of dysregulated pathways across different cell populations.

## Molecular Dynamics Simulations

- 1. All‐atom simulations set-ups and analysis

All-atom (AA) simulations were performed using the CHARMM36m [[28]](https://www.zotero.org/google-docs/?4ATsLe) force field for proteins and lipids, along with the TIP3P [[29]](https://www.zotero.org/google-docs/?wFO0bD) water model. Simulations were run with a time-step of 2 fs and all bonds were constrained using the LINCS algorithm [[30]](https://www.zotero.org/google-docs/?pZ5oSA). Neighbor-searching was accomplished using the Verlet cut-off scheme at every 50 fs. Short-range electrostatic and van der Waals interactions were computed using a 1.2 nm cutoff, using a potential-switch function starting at 1.0 nm. Long-range electrostatic interactions were treated using the Particle Mesh Ewald method [[31]](https://www.zotero.org/google-docs/?77Tk9I). The system temperature was maintained at 310 K using the velocity-rescaling thermostat [[32]](https://www.zotero.org/google-docs/?UH8LGm) with a 0.1 ps coupling constant. Semi-isotropic pressure was applied with the Parrinello-Rahman barostat [[33]](https://www.zotero.org/google-docs/?65ZPle) at 1 bar, using a 1.0 ps coupling constant. The energy minimization step was performed using the steepest descent method, followed by NVT and NPT steps. For each system, three independent simulations were conducted for 300 ns. For the structural analyses, root mean square deviation analysis (RMSD) and root mean square fluctuation (RMSF) were calculated using gmx rmsd and gmx rms commands, respectively, after fitting the proteins to their initial backbone structures using the gmx trjconv command.

- - 1. RAB8b in water

The RAB8b structure was obtained from the AlphaFold Protein Structure Database [[34]](https://www.zotero.org/google-docs/?sFVF8p) (UniProt Q92930). The model shows high per-residue confidence scores (pLDDT), except for the C-terminal region, which displays a highly disordered conformation and lower pLDDT. The protein was inserted in a 10 x 10 x 10 nm simulation box and solvated with TIP3P water molecules (Figure S7A, top). To neutralize the system, counter-ions (K+ and CL-) were added, and position restraints on the protein backbone were applied during minimization, NVT, and NPT equilibration steps.

- - 1. VAMP-3 in a POPC bilayer

The VAMP-3 structure was also obtained from the AlphaFold Protein Structure Database[[34]](https://www.zotero.org/google-docs/?zxgwwg) (UniProt Q15836), with a high overall pLDDT score, excluding the N-terminal region, which exhibited expected disorder due to solvent exposure. The protein/membrane system was built using CHARMM-GUI MembraneBuilder [[35–37]](https://www.zotero.org/google-docs/?J5ERUf). The bilayer contained 72 POPC lipids per leaflet, with one VAMP-3 oriented perpendicularly to the bilayer (Figure S7A, bottom). The system was solvated with 6.5 nm thick water slabs on both sides of the membrane and neutralized with K+ and CL- counter-ions. Position restraints were applied on the protein backbone during minimization, NVT, and NPT equilibration steps.

- 1. Coarse-graining simulations set‐ups and analysis

Coarse-graining (CG) simulations were performed using the Martini3 force field [[38]](https://www.zotero.org/google-docs/?h2bGrh). A time step of 20 fs was used, with neighbor searching updated every 20 steps using the Verlet algorithm. van der Waals interactions we computed with a 1.1 n cutoff, and Coulomb interactions were treated using the reaction-field method with a cutoff of 1.1 nm with a dielectric constant of 15. Semi-isotropic pressure coupling was applied using the C-rescale barostat [[39]](https://www.zotero.org/google-docs/?ZS5fR2) at 1 bar, with a coupling constant of 4.0 ps−1, and compressibility of 3 × 10−4 bar-1. Temperature was maintained at 303K with the velocity-rescaling thermostat [[32]](https://www.zotero.org/google-docs/?Fe8RTc) and a coupling constant of 1.0 ps−1. Energy minimization was performed using the steepest descent method, followed by NVT and NPT equilibration steps. Each system was simulated for a single 10 µs production run. Clustering analyses were performed using gmx clustsize command using a 1.0 nm cut-off and 2D density maps were generated using gmx densmap using a grid size of 0.02 nm.

- - 1. RAB8b and VAMP-3 in a POPC bilayer

CG models of RAB8b and VAMP-3 were obtained using the Martinize2 program [[40]](https://www.zotero.org/google-docs/?x0U9EB), including the -scfix flag [[41]](https://www.zotero.org/google-docs/?sZYd9z). The OLIVES Go̅-like model[[42]](https://www.zotero.org/google-docs/?eUDPzm) was applied to stabilize the protein structures. In the control simulation, 25 VAMP-3 molecules were placed in a 30 x 30 nm POPC bilayer (1400 lipids per leaflet), with their N-terminal regions oriented upward (Figure S7C, right), using the INSANE code [[43]](https://www.zotero.org/google-docs/?PukNEj). For the RAB8b + VAMP-3 system, four RAB8b molecules were positioned at the top edge of the simulation box, away from the VAMP-3 molecules (Figure S7C, left and center). A flat-bottomed position restraint was applied to prevent RAB8b from crossing the simulation boundaries. Both systems were solvated and neutralized with Na+ and Cl- ions.


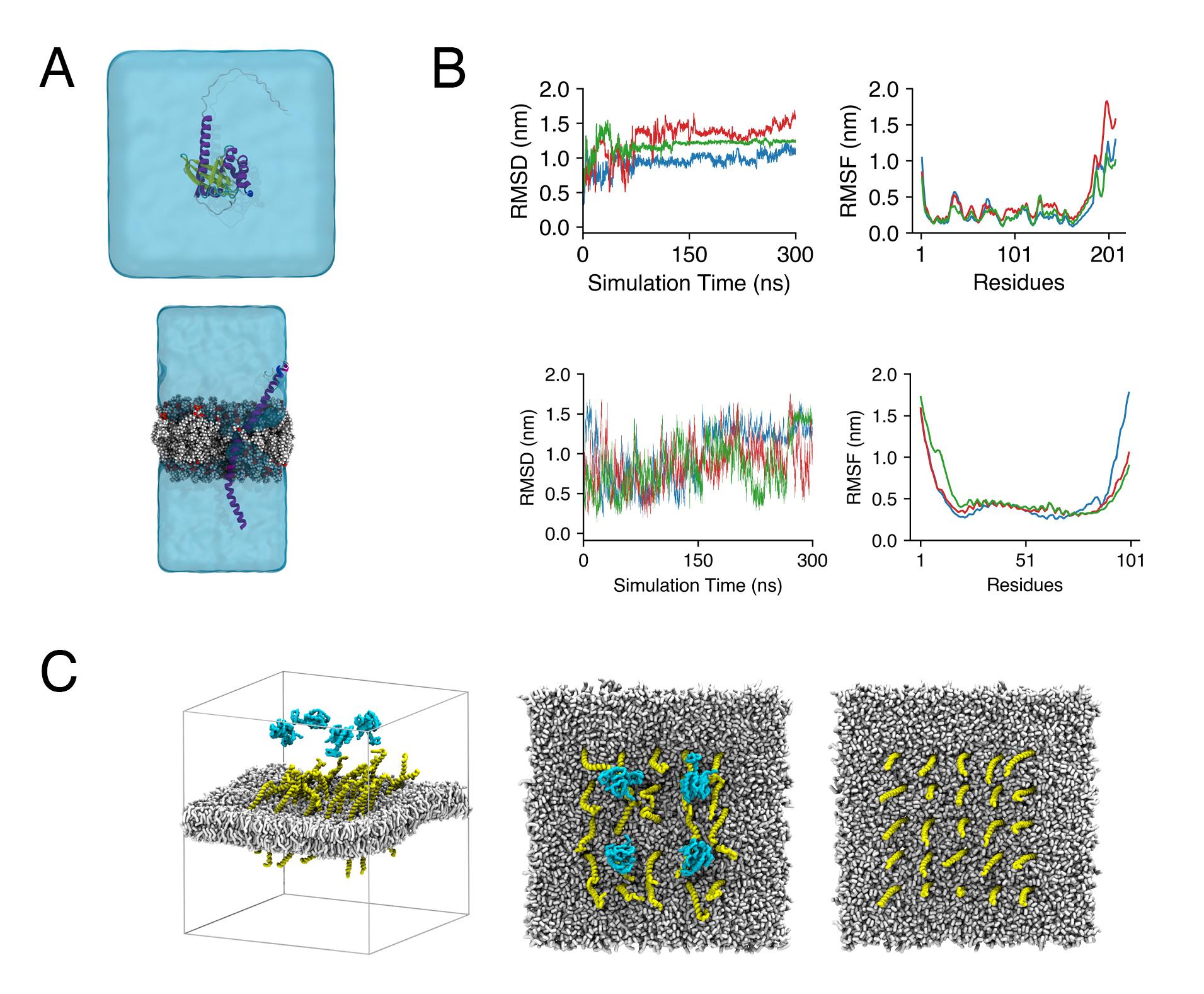


**Figure S7: Atomistic and Coarse-Grained Simulation Setup:** **a.** Snapshots of the initial atomistic systems, RAB8b in water (top) and VAMP-3 in a POPC membrane (bottom). **b.** RMSD and RMSF of the three replica AA simulations (RAB8b on top, VAMP-3 on the bottom). **c.** Snapshots of the CG simulations: RAB8b + VAMP3 in POPC (left and center, side and top view, respectively), and VAMP3 in POPC (right). RAB8b are depicted in cyan, VAMP-3 in yellow, and POPC lipids in silver.

## Coexpression Analysis

For coexpression analysis, the study was initiated by downloading the processed GSE174072 dataset, available from https://covid19.cog.sanger.ac.uk/submissions/release1/blish_awilk_covid_seurat.rds, and subsetting it exclusively to COVID-19 monocytes CD16. To reconstruct pseudobulk expression profiles, raw counts across cell type-samples were aggregated using the 'aggregateExpression' function in the Seurat v4.3.0.1 [[10]](https://www.zotero.org/google-docs/?LPAK6J). . The aggregated counts were then normalized using the regularized log transformation from DESeq2 v1.40.2 [[7]](https://www.zotero.org/google-docs/?75U2ME). Subsequently, the CEMiTool v1.24.0 was employed to construct a signed coexpression network with a minimum of 30 genes per module and an automatically detected power beta[[44]](https://www.zotero.org/google-docs/?LRjB1R). .

For the second coexpression analysis, a Seurat object was created using the 'fraction' method for gene selection, retaining only genes expressed in at least 5% of cells. Metacells were generated by grouping cells according to donor identity, using UMAP for dimensionality reduction and a KNN graph with 25 nearest neighbors. A maximum of 10 cells were allowed to be shared between any two metacells. The resulting aggregated units were processed using default parameters, and a signed coexpression network was constructed using the hdWGCNA package (v0.2.23), with a soft-thresholding power (β) of 9. [[45]](https://www.zotero.org/google-docs/?3kL0Uu).

Lastly, the pySCENIC workflow was applied. Initially, the subset Seurat object for monocytes CD16 was converted into a Loom object using SeuratDisk v0.0.9020. We then inferred a gene regulatory network, generated coexpression modules, and predicted regulon cis targets using the pySCENIC v0.12.1 [[46]](https://www.zotero.org/google-docs/?PjndQt). The auxiliary datasets employed for this analysis included the cis target database (hg38_refseq_r80_v10_db_Gene_based - Homo sapiens - hg38 - refseq_r80 - SCENIC+ databases – Gene-based (aertslab.org)), motif-to-transcription factor annotations (motifs-v9-nr.hgnc-m0.001-o0.0.tbl - Motif2TF annotations (aertslab.org)), and the list of transcription factors (allTFs_hg38.txt - (https://resources.aertslab.org/cistarget/tf_lists/allTFs_hg38.txt)), all retrieved from the Aerts Lab resources. The genes and their corresponding modules can be explored in Table S10.

## Lentiviral shRNA cloning and viral production:

Target sequences to shGFP (5-CAAGCTGACCCTGAAGTTCAT-3), shRAB8B_1 (5’-GTCGTGAAGTTCTAGACAAAT-3’, TRCN0000380248) shRAB8B_2 (5’-CCTGGGTAACAAATGTGATAT-3’, TRCN0000047879) and shOSBPL8 (5’-CAGAGTTCCATCGAATCTATA-3’, TRCN0000146765) were cloned into the AgeI and EcoRI restriction sites of pLKO.1-puro lentiviral vector (Addgene #8453). HEK-293 cell line was used for packing viral particles using psPAX2 (Addgene #12260) and pMD2.G (Addgene #12259) plasmids.

1. Sars-CoV-2 infection:

Immortalized cell line of human colorectal adenocarcinoma CaCo-2 was maintained in DMEM low glucose supplemented with 20% fetal bovine serum and incubated at 37 °C under 5% CO2 in a humidified atmosphere. CaCO-2 cell line was transduced with lentiviral particles (MOI 0.1) from the pLKO.puro shGFP, shRab8b_1, shRab8b_2 or shOsbpl8. After a week of 1 μg/ml puromycin selection, experiments were performed in quadruplicate for each condition of CaCo-2 transduction (shGFP, shRab8b_1, shRab8b_2 and sh), both in the presence and absence of SARS-CoV-2 infection. To infection condition was used SARS-CoV-2 original lineage (GenBank HIAE-02 SARS-CoV- 2/SP02/human/2020/BRA accession number MT126808.1) infection (MOI 0.1) as previously described (Codo et al., 2020). After 24 hours of infection, cells were collected with 200µL of Trizol to total RNA extraction. cDNA reaction was performed using GoScript™ Reverse Transcriptase cDNA (Thermo Scientific - #4311235) synthesis kit according to the manufacturer’s instructions. qPCR reactions were performed to viral load and knockdown confirmation. For viral load a standard curve was generated using serial dilutions of SARS-CoV-2 and Sars-CoV-2 N1 region-specific primers were used (Forward 5'-CAATGCTGCAATCGTGCTAC-3' and Reverse 5'-GTTGCGACTACGTGATGAGG-3'). For knockdown confirmation RAB8B (Forward 5'-TTCCGATGTCGAAAGAATGATCC-3' and Reverse 5'-TGCGCTTGTCTCCAAGAATTTA-3') and OSBPL8 (Forward 5'-TGGCTGATTGGTTAAAGATTCGT-3' and Reverse 5'-GCACCCCAGGTTTCAACACA-3') specific primers were used. All qRT-PCR reactions were performed using QuantiNova SYBR Green PCR Kit (Qiagen - #208056). The controls used in the experiments included shGFP as the control for knockdown assays using shRNA, and non-infected cells as the control for SARS-CoV-2 infection. Statistical analysis was conducted using an unpaired t-test, and differences were considered statistically significant at p-value < 0.05.

# **REFERENCES**

[1.](https://www.zotero.org/google-docs/?oUkqnC)  [Barrett, T.; Wilhite, S.E.; Ledoux, P.; Evangelista, C.; Kim, I.F.; Tomashevsky, M.; Marshall, K.A.; Phillippy, K.H.; Sherman, P.M.; Holko, M.; et al. NCBI GEO: Archive for Functional Genomics Data Sets—Update. *Nucleic Acids Res.* **2013**, *41*, D991–D995, doi:10.1093/nar/gks1193.](https://www.zotero.org/google-docs/?oUkqnC)

[2.](https://www.zotero.org/google-docs/?oUkqnC)  [Chen, S.; Zhou, Y.; Chen, Y.; Gu, J. Fastp: An Ultra-Fast All-in-One FASTQ Preprocessor. *Bioinformatics* **2018**, *34*, i884–i890, doi:10.1093/bioinformatics/bty560.](https://www.zotero.org/google-docs/?oUkqnC)

[3.](https://www.zotero.org/google-docs/?oUkqnC)  [Dobin, A.; Davis, C.A.; Schlesinger, F.; Drenkow, J.; Zaleski, C.; Jha, S.; Batut, P.; Chaisson, M.; Gingeras, T.R. STAR: Ultrafast Universal RNA-Seq Aligner. *Bioinformatics* **2013**, *29*, 15–21, doi:10.1093/bioinformatics/bts635.](https://www.zotero.org/google-docs/?oUkqnC)

[4.](https://www.zotero.org/google-docs/?oUkqnC)  [Babraham Bioinformatics - FastQC A Quality Control Tool for High Throughput Sequence Data Available online: https://www.bioinformatics.babraham.ac.uk/projects/fastqc/ (accessed on 26 October 2022).](https://www.zotero.org/google-docs/?oUkqnC)

[5.](https://www.zotero.org/google-docs/?oUkqnC)  [Ewels, P.; Magnusson, M.; Lundin, S.; Käller, M. MultiQC: Summarize Analysis Results for Multiple Tools and Samples in a Single Report. *Bioinformatics* **2016**, *32*, 3047–3048, doi:10.1093/bioinformatics/btw354.](https://www.zotero.org/google-docs/?oUkqnC)

[6.](https://www.zotero.org/google-docs/?oUkqnC)  [Gonçalves, A.N.A.; Lever, M.; Russo, P.S.T.; Gomes-Correia, B.; Urbanski, A.H.; Pollara, G.; Noursadeghi, M.; Maracaja-Coutinho, V.; Nakaya, H.I. Assessing the Impact of Sample Heterogeneity on Transcriptome Analysis of Human Diseases Using MDP Webtool. *Front. Genet.* **2019**, *10*.](https://www.zotero.org/google-docs/?oUkqnC)

[7.](https://www.zotero.org/google-docs/?oUkqnC)  [Love, M.I.; Huber, W.; Anders, S. Moderated Estimation of Fold Change and Dispersion for RNA-Seq Data with DESeq2. *Genome Biol.* **2014**, *15*, 550, doi:10.1186/s13059-014-0550-8.](https://www.zotero.org/google-docs/?oUkqnC)

[8.](https://www.zotero.org/google-docs/?oUkqnC)  [Durinck, S.; Huber, W.; Davis, S.; Pepin, F.; Buffalo, V.S.; Smith, M. biomaRt: Interface to BioMart Databases (i.e. Ensembl) 2020.](https://www.zotero.org/google-docs/?oUkqnC)

[9.](https://www.zotero.org/google-docs/?oUkqnC)  [Create Elegant Data Visualisations Using the Grammar of Graphics Available online: https://ggplot2.tidyverse.org/ (accessed on 21 October 2023).](https://www.zotero.org/google-docs/?oUkqnC)

[10.](https://www.zotero.org/google-docs/?oUkqnC)  [Hao, Y.; Hao, S.; Andersen-Nissen, E.; Mauck, W.M.; Zheng, S.; Butler, A.; Lee, M.J.; Wilk, A.J.; Darby, C.; Zager, M.; et al. Integrated Analysis of Multimodal Single-Cell Data. *Cell* **2021**, *184*, 3573-3587.e29, doi:10.1016/j.cell.2021.04.048.](https://www.zotero.org/google-docs/?oUkqnC)

[11.](https://www.zotero.org/google-docs/?oUkqnC)  [Stuart, T.; Butler, A.; Hoffman, P.; Hafemeister, C.; Papalexi, E.; Mauck, W.M.; Hao, Y.; Stoeckius, M.; Smibert, P.; Satija, R. Comprehensive Integration of Single-Cell Data. *Cell* **2019**, *177*, 1888-1902.e21, doi:10.1016/j.cell.2019.05.031.](https://www.zotero.org/google-docs/?oUkqnC)

[12.](https://www.zotero.org/google-docs/?oUkqnC)  [Korotkevich, G.; Sukhov, V.; Budin, N.; Shpak, B.; Artyomov, M.N.; Sergushichev, A. Fast Gene Set Enrichment Analysis 2021, 060012.](https://www.zotero.org/google-docs/?oUkqnC)

[13.](https://www.zotero.org/google-docs/?oUkqnC)  [Yates, A.; Akanni, W.; Amode, M.R.; Barrell, D.; Billis, K.; Carvalho-Silva, D.; Cummins, C.; Clapham, P.; Fitzgerald, S.; Gil, L.; et al. Ensembl 2016. *Nucleic Acids Res.* **2016**, *44*, D710–D716, doi:10.1093/nar/gkv1157.](https://www.zotero.org/google-docs/?oUkqnC)

[14.](https://www.zotero.org/google-docs/?oUkqnC)  [Griffith, O.L.; Melck, A.; Jones, S.J.M.; Wiseman, S.M. Meta-Analysis and Meta-Review of Thyroid Cancer Gene Expression Profiling Studies Identifies Important Diagnostic Biomarkers. *J. Clin. Oncol.* **2006**, *24*, 5043–5051, doi:10.1200/JCO.2006.06.7330.](https://www.zotero.org/google-docs/?oUkqnC)

[15.](https://www.zotero.org/google-docs/?oUkqnC)  [Wu, T.; Hu, E.; Xu, S.; Chen, M.; Guo, P.; Dai, Z.; Feng, T.; Zhou, L.; Tang, W.; Zhan, L.; et al. clusterProfiler 4.0: A Universal Enrichment Tool for Interpreting Omics Data. *The Innovation* **2021**, *2*, doi:10.1016/j.xinn.2021.100141.](https://www.zotero.org/google-docs/?oUkqnC)

[16.](https://www.zotero.org/google-docs/?oUkqnC)  [Gu, Z.; Eils, R.; Schlesner, M. Complex Heatmaps Reveal Patterns and Correlations in Multidimensional Genomic Data. *Bioinformatics* **2016**, *32*, 2847–2849, doi:10.1093/bioinformatics/btw313.](https://www.zotero.org/google-docs/?oUkqnC)

[17.](https://www.zotero.org/google-docs/?oUkqnC)  [Nishimura, D. BioCarta Available online: https://www.liebertpub.com/doi/10.1089/152791601750294344 (accessed on 9 January 2024).](https://www.zotero.org/google-docs/?oUkqnC)

[18.](https://www.zotero.org/google-docs/?oUkqnC)  [Huang, R.; Grishagin, I.; Wang, Y.; Zhao, T.; Greene, J.; Obenauer, J.C.; Ngan, D.; Nguyen, D.-T.; Guha, R.; Jadhav, A.; et al. The NCATS BioPlanet – An Integrated Platform for Exploring the Universe of Cellular Signaling Pathways for Toxicology, Systems Biology, and Chemical Genomics. *Front. Pharmacol.* **2019**, *10*.](https://www.zotero.org/google-docs/?oUkqnC)

[19.](https://www.zotero.org/google-docs/?oUkqnC)  [The Gene Ontology Consortium The Gene Ontology Resource: Enriching a GOld Mine. *Nucleic Acids Res.* **2021**, *49*, D325–D334, doi:10.1093/nar/gkaa1113.](https://www.zotero.org/google-docs/?oUkqnC)

[20.](https://www.zotero.org/google-docs/?oUkqnC)  [Liberzon, A.; Birger, C.; Thorvaldsdóttir, H.; Ghandi, M.; Mesirov, J.P.; Tamayo, P. The Molecular Signatures Database (MSigDB) Hallmark Gene Set Collection. *Cell Syst.* **2015**, *1*, 417–425, doi:10.1016/j.cels.2015.12.004.](https://www.zotero.org/google-docs/?oUkqnC)

[21.](https://www.zotero.org/google-docs/?oUkqnC)  [Thomas, P.D.; Ebert, D.; Muruganujan, A.; Mushayahama, T.; Albou, L.-P.; Mi, H. PANTHER: Making Genome-Scale Phylogenetics Accessible to All. *Protein Sci.* **2022**, *31*, 8–22, doi:10.1002/pro.4218.](https://www.zotero.org/google-docs/?oUkqnC)

[22.](https://www.zotero.org/google-docs/?oUkqnC)  [Martens, M.; Ammar, A.; Riutta, A.; Waagmeester, A.; Slenter, D.N.; Hanspers, K.; A. Miller, R.; Digles, D.; Lopes, E.N.; Ehrhart, F.; et al. WikiPathways: Connecting Communities. *Nucleic Acids Res.* **2021**, *49*, D613–D621, doi:10.1093/nar/gkaa1024.](https://www.zotero.org/google-docs/?oUkqnC)

[23.](https://www.zotero.org/google-docs/?oUkqnC)  [Gillespie, M.; Jassal, B.; Stephan, R.; Milacic, M.; Rothfels, K.; Senff-Ribeiro, A.; Griss, J.; Sevilla, C.; Matthews, L.; Gong, C.; et al. The Reactome Pathway Knowledgebase 2022. *Nucleic Acids Res.* **2022**, *50*, D687–D692, doi:10.1093/nar/gkab1028.](https://www.zotero.org/google-docs/?oUkqnC)

[24.](https://www.zotero.org/google-docs/?oUkqnC)  [R: The R Project for Statistical Computing Available online: https://www.r-project.org/ (accessed on 10 June 2023).](https://www.zotero.org/google-docs/?oUkqnC)

[25.](https://www.zotero.org/google-docs/?oUkqnC)  [Federico, A.; Monti, S. hypeR: An R Package for Geneset Enrichment Workflows. *Bioinformatics* **2020**, *36*, 1307–1308, doi:10.1093/bioinformatics/btz700.](https://www.zotero.org/google-docs/?oUkqnC)

[26.](https://www.zotero.org/google-docs/?oUkqnC)  [Bastian, M.; Heymann, S.; Jacomy, M. Gephi: An Open Source Software for Exploring and Manipulating Networks. *Proc. Int. AAAI Conf. Web Soc. Media* **2009**, *3*, 361–362, doi:10.1609/icwsm.v3i1.13937.](https://www.zotero.org/google-docs/?oUkqnC)

[27.](https://www.zotero.org/google-docs/?oUkqnC)  [Cell Line Ontology Available online: https://www.wikidata.org/wiki/Q21039006 (accessed on 23 July 2025).](https://www.zotero.org/google-docs/?oUkqnC)

[28.](https://www.zotero.org/google-docs/?oUkqnC)  [Klauda, J.B.; Venable, R.M.; Freites, J.A.; O’Connor, J.W.; Tobias, D.J.; Mondragon-Ramirez, C.; Vorobyov, I.; MacKerell, A.D.Jr.; Pastor, R.W. Update of the CHARMM All-Atom Additive Force Field for Lipids: Validation on Six Lipid Types. *J. Phys. Chem. B* **2010**, *114*, 7830–7843, doi:10.1021/jp101759q.](https://www.zotero.org/google-docs/?oUkqnC)

[29.](https://www.zotero.org/google-docs/?oUkqnC)  [Price, D.J.; Brooks, C.L., III A Modified TIP3P Water Potential for Simulation with Ewald Summation. *J. Chem. Phys.* **2004**, *121*, 10096–10103, doi:10.1063/1.1808117.](https://www.zotero.org/google-docs/?oUkqnC)

[30.](https://www.zotero.org/google-docs/?oUkqnC)  [LINCS: A Linear Constraint Solver for Molecular Simulations - Hess - 1997 - Journal of Computational Chemistry - Wiley Online Library Available online: https://onlinelibrary.wiley.com/doi/10.1002/(SICI)1096-987X(199709)18:12%3C1463::AID-JCC4%3E3.0.CO;2-H (accessed on 6 February 2025).](https://www.zotero.org/google-docs/?oUkqnC)

[31.](https://www.zotero.org/google-docs/?oUkqnC)  [A Smooth Particle Mesh Ewald Method | The Journal of Chemical Physics | AIP Publishing Available online: https://pubs.aip.org/aip/jcp/article-abstract/103/19/8577/180219/A-smooth-particle-mesh-Ewald-method?redirectedFrom=fulltext (accessed on 6 February 2025).](https://www.zotero.org/google-docs/?oUkqnC)

[32.](https://www.zotero.org/google-docs/?oUkqnC)  [Bussi, G.; Donadio, D.; Parrinello, M. Canonical Sampling through Velocity Rescaling. *J. Chem. Phys.* **2007**, *126*, 014101, doi:10.1063/1.2408420.](https://www.zotero.org/google-docs/?oUkqnC)

[33.](https://www.zotero.org/google-docs/?oUkqnC)  [Parrinello, M.; Rahman, A. Polymorphic Transitions in Single Crystals: A New Molecular Dynamics Method. *J. Appl. Phys.* **1981**, *52*, 7182–7190, doi:10.1063/1.328693.](https://www.zotero.org/google-docs/?oUkqnC)

[34.](https://www.zotero.org/google-docs/?oUkqnC)  [AlphaFold Protein Structure Database: Massively Expanding the Structural Coverage of Protein-Sequence Space with High-Accuracy Models | Nucleic Acids Research | Oxford Academic Available online: https://academic.oup.com/nar/article/50/D1/D439/6430488 (accessed on 6 February 2025).](https://www.zotero.org/google-docs/?oUkqnC)

[35.](https://www.zotero.org/google-docs/?oUkqnC)  [Wu, E.L.; Cheng, X.; Jo, S.; Rui, H.; Song, K.C.; Dávila-Contreras, E.M.; Qi, Y.; Lee, J.; Monje-Galvan, V.; Venable, R.M.; et al. CHARMM-GUI Membrane Builder toward Realistic Biological Membrane Simulations. *J. Comput. Chem.* **2014**, *35*, 1997–2004, doi:10.1002/jcc.23702.](https://www.zotero.org/google-docs/?oUkqnC)

[36.](https://www.zotero.org/google-docs/?oUkqnC)  [Jo, S.; Kim, T.; Iyer, V.G.; Im, W. CHARMM-GUI: A Web-Based Graphical User Interface for CHARMM. *J. Comput. Chem.* **2008**, *29*, 1859–1865, doi:10.1002/jcc.20945.](https://www.zotero.org/google-docs/?oUkqnC)

[37.](https://www.zotero.org/google-docs/?oUkqnC)  [CHARMM-GUI Membrane Builder for Mixed Bilayers and Its Application to Yeast Membranes: Biophysical Journal Available online: https://www.cell.com/biophysj/fulltext/S0006-3495(09)00791-7?_returnURL=https%3A%2F%2Flinkinghub.elsevier.com%2Fretrieve%2Fpii%2FS0006349509007917%3Fshowall%3Dtrue (accessed on 6 February 2025).](https://www.zotero.org/google-docs/?oUkqnC)

[38.](https://www.zotero.org/google-docs/?oUkqnC)  [Martini 3: A General Purpose Force Field for Coarse-Grained Molecular Dynamics | Nature Methods Available online: https://www.nature.com/articles/s41592-021-01098-3 (accessed on 6 February 2025).](https://www.zotero.org/google-docs/?oUkqnC)

[39.](https://www.zotero.org/google-docs/?oUkqnC)  [Pressure Control Using Stochastic Cell Rescaling | The Journal of Chemical Physics | AIP Publishing Available online: https://pubs.aip.org/aip/jcp/article-abstract/153/11/114107/199610/Pressure-control-using-stochastic-cell-rescaling?redirectedFrom=fulltext (accessed on 6 February 2025).](https://www.zotero.org/google-docs/?oUkqnC)

[40.](https://www.zotero.org/google-docs/?oUkqnC)  [Kroon, P.C.; Grunewald, F.; Barnoud, J.; Tilburg, M. van; Souza, P.C.T.; Wassenaar, T.A.; Marrink, S.J. Martinize2 and Vermouth: Unified Framework for Topology Generation. *eLife* **2023**, *12*, doi:10.7554/eLife.90627.1.](https://www.zotero.org/google-docs/?oUkqnC)

[41.](https://www.zotero.org/google-docs/?oUkqnC)  [Herzog, F.A.; Braun, L.; Schoen, I.; Vogel, V. Improved Side Chain Dynamics in MARTINI Simulations of Protein–Lipid Interfaces. *J. Chem. Theory Comput.* **2016**, *12*, 2446–2458, doi:10.1021/acs.jctc.6b00122.](https://www.zotero.org/google-docs/?oUkqnC)

[42.](https://www.zotero.org/google-docs/?oUkqnC)  [Pedersen, K.B.; Borges-Araújo, L.; Stange, A.D.; Souza, P.C.T.; Marrink, S.J.; Schiøtt, B. OLIVES: A Go̅-like Model for Stabilizing Protein Structure via Hydrogen Bonding Native Contacts in the Martini 3 Coarse-Grained Force Field. *J. Chem. Theory Comput.* **2024**, *20*, 8049–8070, doi:10.1021/acs.jctc.4c00553.](https://www.zotero.org/google-docs/?oUkqnC)

[43.](https://www.zotero.org/google-docs/?oUkqnC)  [Wassenaar, T.A.; Ingólfsson, H.I.; Böckmann, R.A.; Tieleman, D.P.; Marrink, S.J. Computational Lipidomics with Insane: A Versatile Tool for Generating Custom Membranes for Molecular Simulations. *J. Chem. Theory Comput.* **2015**, *11*, 2144–2155, doi:10.1021/acs.jctc.5b00209.](https://www.zotero.org/google-docs/?oUkqnC)

[44.](https://www.zotero.org/google-docs/?oUkqnC)  [Russo, P.S.T.; Ferreira, G.R.; Cardozo, L.E.; Bürger, M.C.; Arias-Carrasco, R.; Maruyama, S.R.; Hirata, T.D.C.; Lima, D.S.; Passos, F.M.; Fukutani, K.F.; et al. CEMiTool: A Bioconductor Package for Performing Comprehensive Modular Co-Expression Analyses. *BMC Bioinformatics* **2018**, *19*, 56, doi:10.1186/s12859-018-2053-1.](https://www.zotero.org/google-docs/?oUkqnC)

[45.](https://www.zotero.org/google-docs/?oUkqnC)  [Morabito, S.; Reese, F.; Rahimzadeh, N.; Miyoshi, E.; Swarup, V. hdWGCNA Identifies Co-Expression Networks in High-Dimensional Transcriptomics Data. *Cell Rep. Methods* **2023**, *3*, doi:10.1016/j.crmeth.2023.100498.](https://www.zotero.org/google-docs/?oUkqnC)

[46.](https://www.zotero.org/google-docs/?oUkqnC)  [Van De Sande, B.; Flerin, C.; Davie, K.; De Waegeneer, M.; Hulselmans, G.; Aibar, S.; Seurinck, R.; Saelens, W.; Cannoodt, R.; Rouchon, Q.; et al. A Scalable SCENIC Workflow for Single-Cell Gene Regulatory Network Analysis. *Nat. Protoc.* **2020**, *15*, 2247–2276, doi:10.1038/s41596-020-0336-2.](https://www.zotero.org/google-docs/?oUkqnC)
